# Overall survival of palbociclib plus endocrine therapy in Japanese patients with HR+/HER2– advanced breast cancer in the first-or second-line setting: a multicenter observational study (P-BRIDGE study)

**DOI:** 10.1007/s12282-025-01689-4

**Published:** 2025-04-05

**Authors:** Shigenori E. Nagai, Masaya Hattori, Tetsuhiro Yoshinami, Hiroko Masuda, Takuho Okamura, Kenichi Watanabe, Takahiro Nakayama, Michiko Tsuneizumi, Daisuke Takabatake, Michiko Harao, Hiroshi Yoshino, Natsuko Mori, Hiroyuki Yasojima, Chiya Oshiro, Madoka Iwase, Miki Yamaguchi, Takafumi Sangai, Shinsuke Sasada, Takanori Ishida, Manabu Futamura, Yasuaki Muramatsu, Nobuyoshi Kosaka, Norikazu Masuda

**Affiliations:** 1 Division of Breast Oncology, Saitama Prefectural Cancer Center, Saitama, Japan; 2 Department of Breast Oncology, Aichi Cancer Center, Nagoya, Japan; 3 Department of Breast and Endocrine Surgery, Graduate School of Medicine, Osaka University, Osaka, Japan; 4 Department of Breast Surgical Oncology, School of Medicine, Showa University, Tokyo, Japan; 5 Department of Breast Oncology, Tokai University School of Medicine, Tokyo, Japan; 6 Department of Breast Surgery, National Hospital Organization Hokkaido Cancer Center, Sapporo, Japan; 7 Department of Breast and Endocrine Surgery, Osaka International Cancer Institute, Osaka, Japan; 8 Department of Breast Surgery, Shizuoka General Hospital, Shizuoka, Japan; 9 Department of Breast Oncology, National Hospital Organization Shikoku Cancer Center, Matsuyama, Japan; 10 Department of Breast Oncology, Jichi Medical University, Shimotsuke, Japan; 11 Breast and Endocrinological Surgery, Ishikawa Prefectural Central Hospital, Kanazawa, Japan; 12 Department of Breast Surgery, Seirei Hamamatsu General Hospital, Hamamatsu, Japan; 13 Department of Surgery, Breast Oncology, National Hospital Organization Osaka National Hospital, Osaka, Japan; 14 Department of Breast Surgery, Kaizuka City Hospital, Osaka, Japan; 15 Department of Breast and Endocrine Surgery, Nagoya University Hospital, Nagoya, Japan; 16 Department of Breast Surgery, JCHO Kurume General Hospital, Kurume, Japan; 17 Department of Breast and Thyroid Surgery, Kitasato University School of Medicine, Sagamihara, Japan; 18 Department of Surgical Oncology, Research Institute for Radiation Biology and Medicine, Hiroshima University, Hiroshima, Japan; 19 Division of Breast and Endocrine Surgical Oncology, Tohoku University Graduate School of Medicine, Tokyo, Japan; 20 Department of Breast Surgery, Gifu University Hospital, Gifu, Japan; 21 Oncology Medical Affairs, Pfizer Japan Inc, Tokyo, Japan; 22 Department of Breast Surgery, Graduate School of Medicine, Kyoto University, 54 Shogoin-Kawahara-Cho, Sakyo-Ku, Kyoto, 606-8507 Japan

**Keywords:** Advanced breast cancer, CDK4/6 inhibitors, Palbociclib, Real-world evidence, Overall survival, Japanese patients

## Abstract

**Background:**

Recently, we reported the real-world effectiveness of palbociclib plus endocrine therapy (ET) in HR+/HER2– advanced breast cancer (ABC) in Japan (NCT05399329). However, median overall survival (OS) was not reached because of limited follow-up (36 months). Here, we present follow-up data from this study, including real-world clinical outcomes and treatment patterns.

**Methods:**

The P-BRIDGE study was a multi-center, observational study evaluating the real-world effectiveness and treatment patterns of patients diagnosed with HR+/HER2– ABC who received palbociclib plus ET in first (1L) or second line (2L) in Japan. The primary endpoint was real-world progression-free survival (rwPFS); secondary endpoints included OS and chemotherapy-free survival (CFS).

**Results:**

Of the 693 eligible patients, 426 and 267 patients received palbociclib with ET as 1L and 2L treatment, respectively. After a median follow-up of 48.1 months, the median rwPFS (95% CI) was 26.2 months (21.4-30.4) for 1L and 14.9 months (11.7-18.3) for 2L, respectively. Median OS (95% CI) was 68.2 months (60.8-NE) for 1L and 50.7 months (42.2-57.2) for 2L, respectively. OS analysis was also performed in the following subgroups: TFI < 12 months/TFI ≥ 12months/de novo metastatic median OS was 56.3 months (43.9-68.2), NR (NE-NE), NR (56.3-NE), visceral metastasis was 65.0 months (56.3-NE), liver metastasis was 46.4 months (37.2-NE), and bone only metastasis was NR (57.8-NE) in 1L, respectively.

**Conclusions:**

The updated results from this study further confirm the real-world effectiveness of palbociclib plus ET in routine clinical practice in Japan. More than 5 years of median OS in 1L was observed, supporting the use of palbociclib plus ET as 1L standard of care for HR+/HER2– ABC.

**Supplementary Information:**

The online version contains supplementary material available at 10.1007/s12282-025-01689-4.

## Introduction

Palbociclib, a selective, oral inhibitor of the cyclin-dependent kinases 4 and 6 (CDK4/6), blocks G1 to S phase cell cycle progression to prevent uncontrolled cell proliferation [1, 2]. In the phase III PALOMA-2 and -3 trials, first- or subsequent-line palbociclib plus endocrine therapy (ET) treatment significantly improved progression-free survival (PFS) compared with ET alone in patients with hormone receptor positive (HR+)/human epidermal growth factor receptor 2 negative (HER2–) advanced breast cancer (ABC) [3–5]. Palbociclib was approved in the United States in combination with letrozole in February 2015 for women with HR+/HER2– ABC [6]. In Japan, palbociclib with ET was approved in September 2017 for the treatment of inoperable or recurrent breast cancer [7–10].

Despite the PFS benefits reported with palbociclib in randomized controlled trials (RCTs), no statistically significant difference was shown in the secondary endpoint of overall survival (OS). In PALOMA-2, median OS was 53.9 months [95% CI 49.8-60.8] in the palbociclib + letrozole arm versus 51.2 months [95% CI: 43.7-58.9] in the placebo + letrozole arm (hazard ratio [HR] [95% CI]: 0.96 [0.78-1.18]; *p* = 0.34) [11]. A numerical survival advantage was reported in favour of palbociclib in PALOMA-3, with a median OS of 34.9 months (95% CI: 28.8, 40.0) with palbociclib + fulvestrant versus 28.0 months (95% CI: 23.6, 34.6) in the placebo + fulvestrant group, although the difference in OS was not statistically significant (HR: 0.81 [95% CI: 0.64, 1.03]; p = 0.09) [12].

It is known that RCTs are subject to strict eligibility criteria, limiting the potential reach of patients. Large-scale, real-world data from the US (P-REALITY and P-REALITY X) demonstrated the effectiveness of palbociclib in HR+/HER2– ABC patients in routine clinical practice [13, 14]. P-REALITY [13] and P-REALITY X [14] studies both showed longer OS in the palbociclib group versus the letrozole alone or aromatase inhibitor alone group (HR: 0.66 [95% CI: 0.53, 0.82] and HR: 0.76 [95% CI: 0.65, 0.87], respectively). However, local clinical practice and available medical treatment in Japan may vary from those in Western countries. Further, there is no reliable, large-scale database like Flatiron in Japan. We recently reported the interim analysis results of a real-world, multi-center, observational cohort study to evaluate real-world effectiveness of palbociclib in HR+/HER2– ABC patients in Japan; P-BRIDGE study (Palbociclib in Japan: Breast cancer Real-world Investigation of DruG utilization and Effectiveness; NCT05399329) [15]. At the interim analysis, real-world PFS (rwPFS) was 24.5 months for first-line (1L) and 14.5 months for second-line (2L) treatment groups with median follow-up of 38.3 months; however, the median OS could not be determined, partly because the number of events was insufficient for OS data [15]. Due to the differences between Western and Japanese clinical practice, patient characteristics, and treatment environments, including insurance systems and available drugs, OS data from Japanese clinical practice is of great clinical relevance for Japanese physicians to obtain insights on the expected OS for the patients in front of them, to better understand the efficacy profile of palbociclib, and to ensure its appropriate use. Here, we report the updated analysis results of P-BRIDGE study including rwPFS, OS, and chemotherapy-free survival (CFS) with median follow-up of 48.1 months.

## Methods

### Study design and data source

The P-BRIDGE follow-up study was a multi-center, observational study (NCT05399329) conducted in Japan. A medical record chart abstraction approach leveraging a uniform case report-form was utilized to capture the real-world treatment outcomes and treatment patterns of patients with ABC who have received palbociclib plus ET as first- or second-line setting from 20 study sites across the country.

This study was approved by the Institutional Review Board of Nagoya University and was conducted according to the Ethical Guidelines for Medical and Health Research Involving Human Subjects issued by the Minister of Health, Labour, and Welfare (MHLW), the Declaration of Helsinki, and as per the other legal and regulatory requirements. Patients provided written informed consent prior to study participation.

## Patients

All patients who initiated palbociclib plus ET across eligible study sites from 15 December 2017 to 31 December 2020 were screened. Study site feasibility was assessed as per the criteria specified in Yoshinami et al. Breast Cancer 2024 [15].

Patients were eligible if they were aged ≥ 20 years, had a diagnosis of HR+/HER2− ABC, and had received palbociclib plus ET in the 1L or 2L setting, defined according to the Japanese Breast Cancer Society Clinical Practice Guidelines for systemic treatment of breast cancer, 2018 edition [16]. Patients with medical records for > 6 months from palbociclib initiation were included regardless of palbociclib continuation. Patients with medical records for < 6 months from palbociclib initiation were still included if they had any specific event recorded (death, progressive disease [PD], or palbociclib discontinuation due to adverse events [AEs]).

Patients were excluded if they had previously received chemotherapy (CT) as 1L treatment. One induction CT regimen was permitted if the purpose of the regimen was to reduce initial tumor burden, and the patients were switched to ET before PD. Patients were also excluded if they had previously participated in, or were participating in, any interventional clinical trial.

## Outcomes

The primary endpoint of the study was rwPFS of palbociclib plus ET as 1L and 2L treatment. rwPFS was defined as the time from the start of palbociclib plus ET to physician-documented PD or death due to any cause, whichever occurred first.

Secondary endpoints included OS as 1L and 2L treatment, CFS, baseline demographics, clinical characteristics, and treatment patterns (initial dose, dose reduction and reasons, treatment discontinuation and reasons, type of subsequent therapy). OS was defined as the time from the start of palbociclib treatment to death due to any cause. CFS was defined as the time from start of palbociclib treatment to the start of first subsequent chemotherapy or death due to any cause, whichever occurred first.

## Statistical analysis

Two analysis sets were analysed: all patients who started palbociclib at 125 mg/day, which is the starting dose employed in RCTs and also the recommended dose in the package insert [3, 4, 17], as well as all patients irrespective of starting dose of palbociclib to evaluate real-world effectiveness in clinical practice in Japan. All the outcomes were evaluated by treatment lines. Subgroup analyses evaluated the effect of visceral metastasis (including lung, liver, pleural effusion, ascites, ovary, uterus, adrenal gland, kidney, spleen, digestive tract, and thyroid), liver metastasis, bone-only metastasis, and treatment-free interval (TFI). TFI was defined as the time from the end of adjuvant therapy to the diagnosis date of recurrence.

Continuous variables were summarized descriptively, including means, standard deviations, medians, and ranges. Categorical variables were summarized using frequencies and proportions. Time to event analyses, including rwPFS, OS, and CFS, were assessed using the Kaplan-Meier method, with medians and 95% CIs presented.

All statistical analyses were performed using SAS® version 9.4 (SAS Institute, Cary, NC, USA).

## Results

### Demographic and baseline disease characteristics of patients

**Fig. 1:**
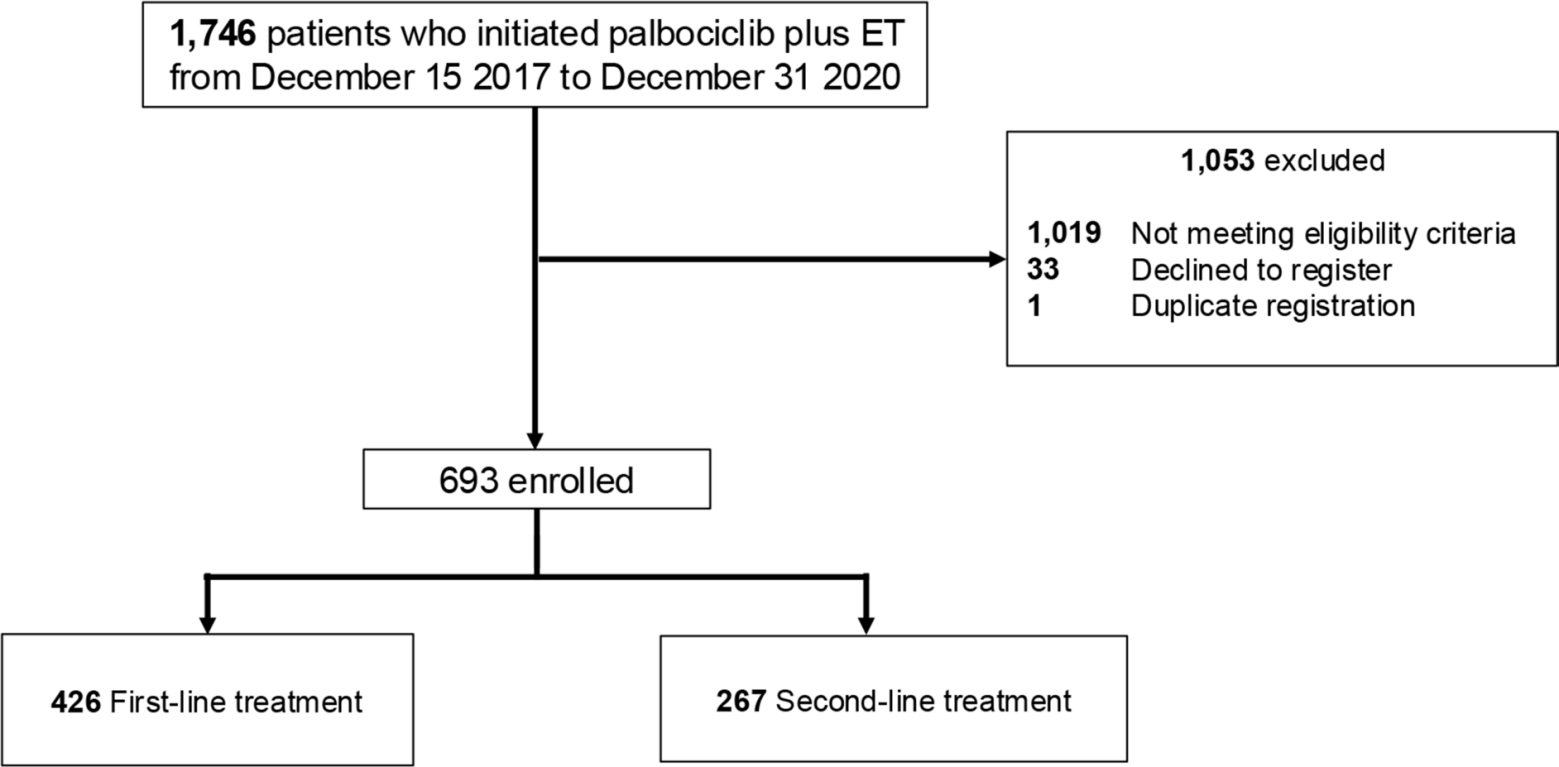
Patient flow diagram

A total of 1,746 patients, who initiated palbociclib treatment regardless of treatment line between December 15, 2017 and December 31, 2020, were screened. Of these, 1,053 patients were excluded (Fig. 1) for the following reasons: screen fail, mainly due to the treatment line being third line or later (n = 1019), declined to register (n = 33), and duplicate registration (n = 1).

Overall, 693 patients met the eligibility criteria (Fig. 1), including 426 receiving 1L and 267 receiving 2L treatment of palbociclib plus ET, respectively. In this updated analysis, the median follow-up time from palbociclib initiation was 48.1 months.

Demographic and clinical characteristics of patients receiving palbociclib plus ET as 1L and 2L treatment are presented in Table 1. In the 1L and 2L treatment groups, respectively, the median (min-max) age was 60 (29-87) and 60 (32-87) years. 78.9% and 80.9% had a favourable ECOG performance status (PS) score of 0-1 (18.3 and 18.0% with missing information, respectively). Visceral and liver metastases were present in 50.2% and 16.9% of patients in the 1L treatment group and 59.9% and 27.3% of patients in the 2L treatment group, respectively. Bone only metastases were present in 24.6% of patients in the 1L treatment group and 18.7% of patients in the 2L treatment group. Furthermore, in the 1L treatment group, 45.8%/20.4%/26.1% of patients had TFI < 12 months, TFI ≥ 12 months, and de novo metastasis, respectively. A total of 50.9% of patients in the 1L treatment group and 34.5% of patients in the 2L treatment group had symptoms present at palbociclib initiation (symptoms included bone pain, shortness of breath, coughing, headaches, dizziness, nausea, swelling around the neck and armpits, numbness in the limbs, abdominal bloating, and jaundice).

**Table 1:** Patient demographics and clinical characteristics

	1L (n = 426)	2L (n = 267)
Age (years), median (range)	60.0 (29.0, 87.0)	60.0 (32.0, 87.0)
Age category (years), n (%)		
≤ 49	88 (20.7)	60 (22.5)
50–64	178 (41.8)	101 (37.8)
65–74	118 (27.7)	66 (24.7)
≥ 75	42 (9.9)	40 (15.0)
Sex, n (%)		
Male	3 (0.7)	1 (0.4)
Female	423 (99.3)	266 (99.6)
Menopausal status, n (%) ^a^		
Pre/perimenopausal	85 (20.1)	69 (25.9)
Postmenopausal	302 (71.4)	180 (67.7)
Unknown	36 (8.5)	17 (6.4)
Stage at the time of initial treatment, n (%)		
0	2 (0.5)	2 (0.7)
I	58 (13.6)	34 (12.7)
II	189 (44.4)	104 (39.0)
III	70 (16.4)	42 (15.7)
IV	99 (23.2)	72 (27.0)
Unknown	8 (1.9)	13 (4.9)
ECOG PS, n (%)		
0	269 (63.1)	153 (57.3)
1	67 (15.7)	63 (23.6)
≥ 2	12 (2.8)	3 (1.1)
Unknown	78 (18.3)	48 (18.0)
Disease sites, n (%)		
Visceral metastasis	214 (50.2)	160 (59.9)
Liver metastasis	72 (16.9)	73 (27.3)
Bone-only metastasis	105 (24.6)	50 (18.7)
DFI, n (%)^b^		
≥ 24 months	278 (65.3)	165 (61.8)
< 24 months	39 (9.2)	23 (8.6)
TFI, n (%) ^c^		
De novo metastasis/others	111 (26.1)	75 (28.1)
≥ 12 months	87 (20.4)	54 (20.2)
< 12 months	195 (45.8)	105 (39.3)
Symptoms at the start of palbociclib, n (%)^d^		
Yes	217 (50.9)	92 (34.5)
No	191 (44.8)	163 (61.0)
Unknown	18 (4.2)	12 (4.5)
Prior ET for (neo)adjuvant, n (%)	291 (68.3)	176 (65.9)
Prior CT for (neo)adjuvant, n (%)	217 (50.9)	125 (46.8)
Induction CT, n (%)	23 (5.4)	8 (3.0)

*ABC* advanced breast cancer, *CT* chemotherapy, *DFI* disease-free interval (the time from the date of breast cancer surgery to the diagnosis date of recurrence), *ECOG PS* Eastern Cooperative Oncology Group performance status, *ET* endocrine therapy, *TFI* treatment-free interval (the time from the end of adjuvant therapy to the diagnosis date of recurrence)

^a^The denominator is the number of female patients

^b^Percentage was calculated based on patients with disease stage other than “stage IV”. The patients without the date of breast cancer surgery were excluded from this calculation

^c^“Others” included patients who had surgery but did not undergo adjuvant therapy. The patients without the date of breast cancer surgery were excluded from this calculation

^d^Symptoms included bone pain, shortness of breath, coughing, headaches, dizziness, nausea, swelling around the neck and armpits, numbness in the limbs, abdominal bloating, and jaundice

### Real-world treatment pattern and dose modification of palbociclib

In the 1L and 2L treatment setting, 90.4% and 87.3% of patients initiated palbociclib at a dose of 125 mg/day, respectively (Table 2). 78.2% of patients in 1L and 88.0% of patients in 2L had discontinued palbociclib treatment, with the most common reason being disease progression, and adverse events. Dose reductions occurred in 74.4% in 1L and 70.8% in 2L. Among the patients who reduced the dose of palbociclib, 45.8% in 1L and 41.6% in 2L of patients received a dose of 75 mg/day. Fulvestrant was the most frequent ET combination with palbociclib (56.3% and 77.2% in 1L and 2L, respectively), with 38.0% and 16.1% of patients receiving letrozole in these two setting (Table 2).

**Table 2:** Treatment pattern and dose modification of palbociclib

	1L (n = 426) n (%)	2L (n = 267) n (%)
Initial palbociclib dose (mg/day)		
125	385 (90.4)	233 (87.3)
100	33 (7.7)	28 (10.5)
75	8 (1.9)	5 (1.9)
Other	0 (0)	1 (0.4)
Status of palbociclib administration^a^		
Ongoing	93 (21.8)	32 (12.0)
Discontinued	333 (78.2)	235 (88.0)
Reason for discontinuation of palbociclib^b,c^		
Disease progression	231 (69.4)	182 (77.4)
Adverse events	70 (21.0)	41 (17.4)
Other	39 (11.7)	17 (7.2)
Patients requiring dose reduction^d^		
No	109 (25.6)	78 (29.2)
Yes	317 (74.4)	189 (70.8)
100 (mg/day)	106 (24.9)	71 (26.6)
75 (mg/day)	195 (45.8)	111 (41.6)
Other (mg/day)	16 (3.8)	7 (2.6)
Timing of first dose reduction of palbociclib, n (%)^d^		
≤ 3 months	249 (78.5)	152 (80.4)
3– ≤ 6 months	34 (10.7)	17 (9.0)
6– ≤ 9 months	10 (3.2)	6 (3.2)
> 9 months	24 (7.6)	14 (7.4)
Endocrine therapy in combination with palbociclib^e^		
Fulvestrant	240 (56.3)	206 (77.2)
Letrozole	162 (38.0)	43 (16.1)
Anastrozole	17 (4.0)	13 (4.9)
Exemestane	2 (0.5)	3 (1.1)
Tamoxifen	7 (1.6)	3 (1.1)

^a^Data cutoff date was February 16, 2024

^b^Percentage was calculated with patients who discontinued palbociclib

^c^The different reasons for palbociclib discontinuation in the same patient were counted in the respective group

^d^Percentage was calculated with patients who underwent dose reduction

^e^The different endocrine therapies used in the same patient within the same treatment line were counted in the respective group

In this analysis, 21.8% of 1L patients (93/426) and 12.0% of 2L patients (32/267) were still receiving palbociclib combination therapy. 69.2% (295/426) of 1L patients and 77.9% (208/267) of 2L patients transitioned to a subsequent therapy, while 8.9% (38/426) of 1L patients and 10.1% (27/267) of 2L patients terminated study treatment without subsequent therapy. Among the 295 patients who transitioned to a subsequent therapy after 1L palbociclib, 61.7% were treated with endocrine-based therapies, including ET + CDK4/6i (palbociclib or abemaciclib; 24.4%), as the subsequent 2L therapy. Among the 208 patients who transitioned to a subsequent therapy after 2L palbociclib, 62.0% were treated with endocrine-based therapies, including ET + CDK4/6i (16.8%), as the subsequent 3L therapy (Table 3).

**Table 3:** Type of subsequent therapy

	1L	2L
	(N = 426)	(N = 267)
	n (%)	n (%)
No subsequent therapy received	131 (30.8)	59 (22.1)
Study treatment ongoing	93 (21.8)	32 (12.0)
Study treatment terminated	38 (8.9)	27 (10.1)
Received first subsequent therapy^a^	295 (69.2)	208 (77.9)
Endocrine-based therapy	182 (61.7)	129 (62.0)
Endocrine therapy (ET) alone	53 (18.0)	33 (15.9)
ET + CDK4/6 inhibitor^b^	72 (24.4)	35 (16.8)
ET + everolimus	55 (18.6)	61 (29.3)
ET + other	2 (0.7)	0 (0.0)
Chemotherapy (CT)	101 (34.2)	76 (36.5)
CT + bevacizumab	35 (11.9)	14 (6.7)
CT (oral fluoropyrimidine)	43 (14.6)	46 (22.1)
CT (excluding oral fluoropyrimidine)	23 (7.8)	16 (7.7)
Other	12 (4.1)	3 (1.4)

^a^Subsequent percentages are calculated based on number of patients receiving first subsequent therapy

^b^Patients received endocrine therapy in combination with a CDK4/6 inhibitor (palbociclib or abemaciclib)

## Overall real-world PFS and OS

**Fig. 2:**
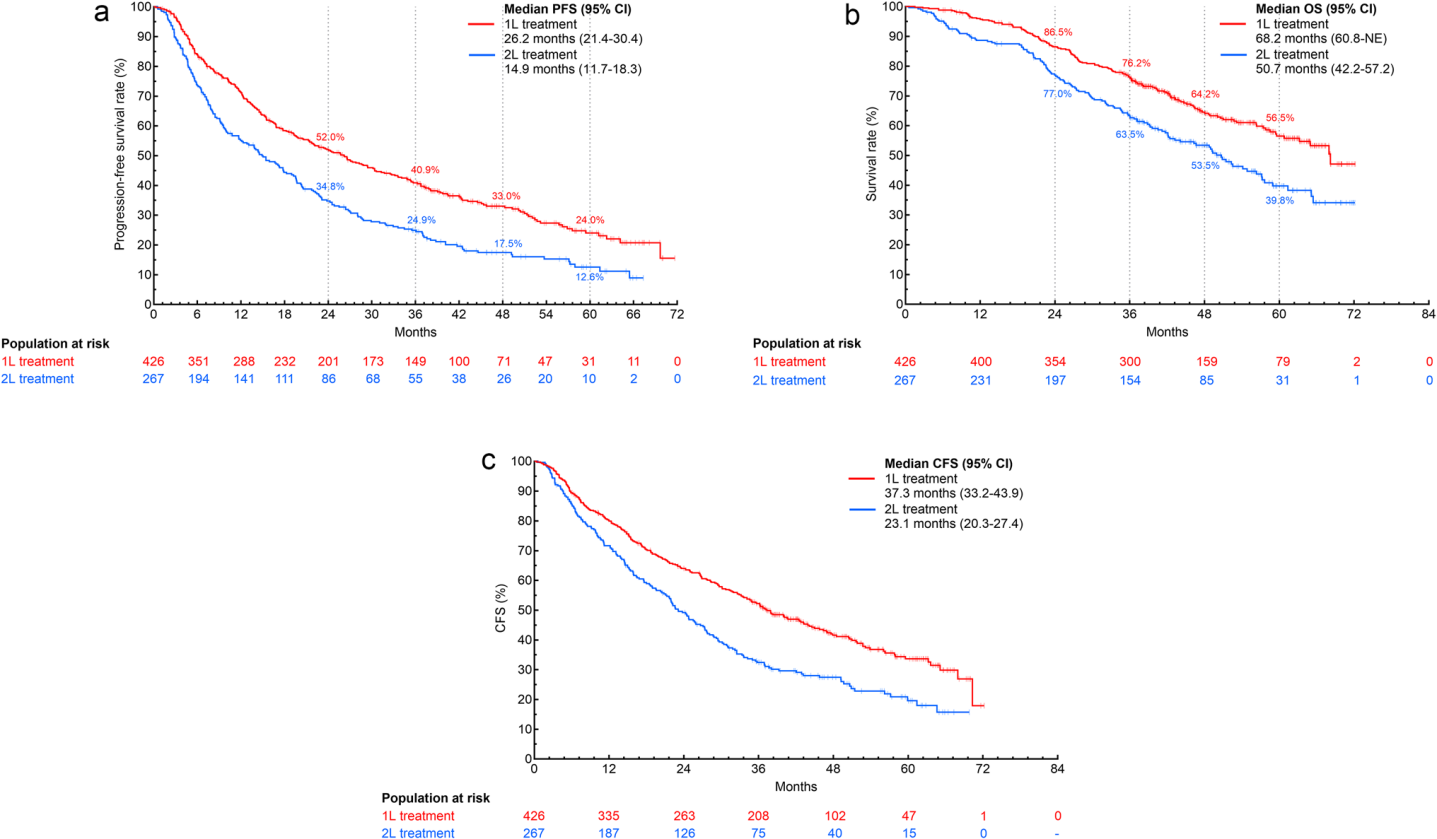
**A** Real-world PFS **B** OS and **C** CFS of palbociclib plus ET as 1L and 2L treatment. *PFS* progression-free survival, *OS* overall survival, *CFS* chemotherapy-free survival, *ET* endocrine therapy

Median rwPFS (95% CI) was 26.2 months (21.4-30.4) for the 1L and 14.9 months (11.7-18.3) for the 2L treatment group (Fig. 2A). Median OS (95% CI) was 68.2 months (60.8-not estimated (NE)) for 1L and 50.7 months (42.2-57.2) for 2L (Fig. 2B). Survival rates at 24, 36, 48, and 60 months were 86.5%, 76.2%, 64.2%, and 56.5%, respectively, in patients receiving palbociclib as 1L treatment. Corresponding rates were 77.0%, 63.5%, 53.5%, and 39.8% in patients receiving palbociclib as 2L treatment. Median CFS (95% CI) was 37.3 months (33.2-43.9) for 1L and 23.1 months (20.3-27.4) for 2L (Fig. 2C).

### Subgroup analysis of rwPFS and OS by the presence/absence of visceral, liver and bone metastasis, and by treatment-free interval

**Fig. 3:**
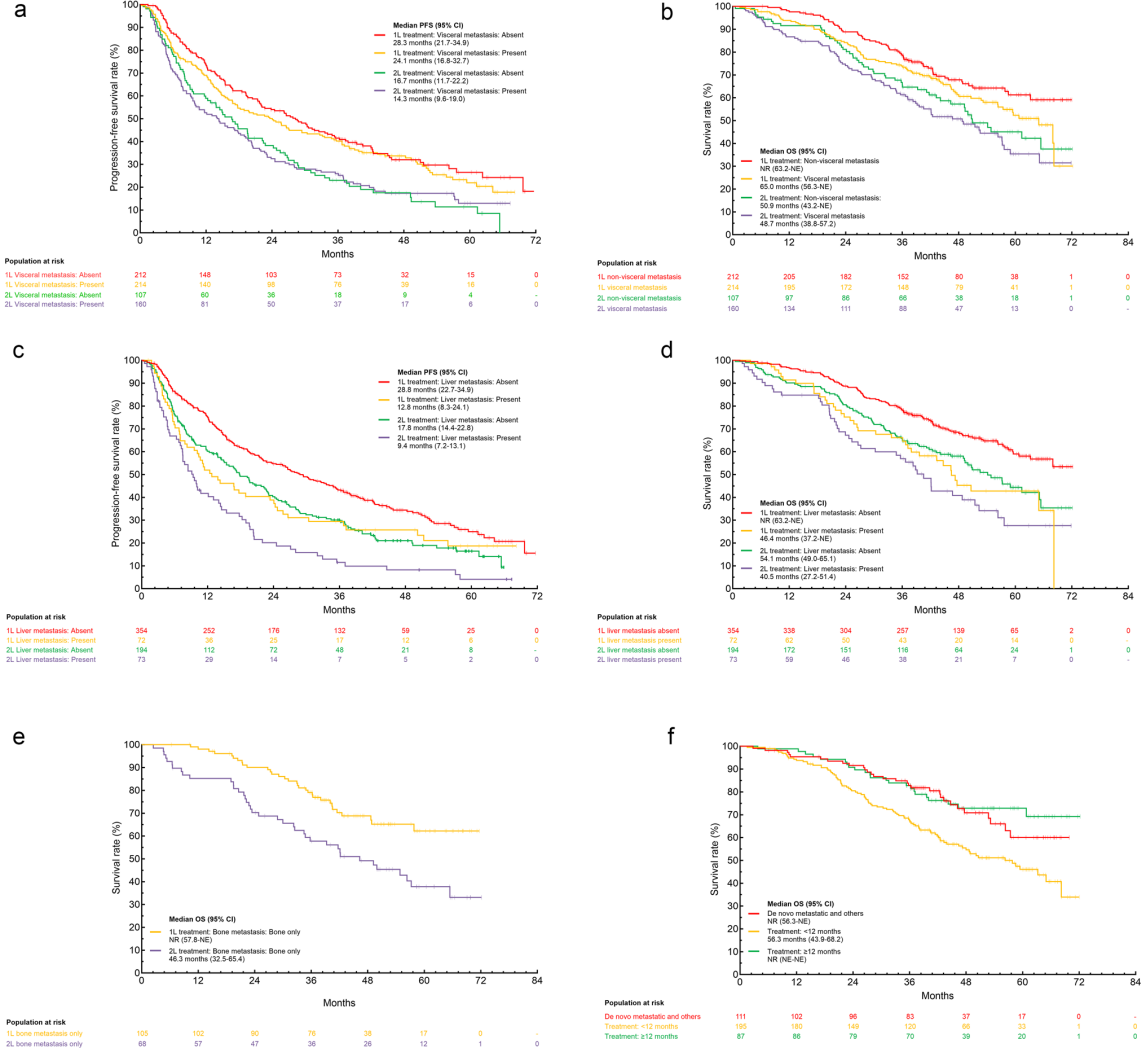
Analysis for rwPFS and OS of palbociclib plus ET as 1L and 2L treatment in subgroup. rwPFS (**A**) and OS (**B**) in the patients of absence or presence of visceral metastasis. rwPFS (**C**) and OS (**D**) in the patients of absence or presence of liver metastasis. OS (**E**) in the patients with bone only metastasis. OS (F) in patients with TFI ≥ 12 months, TFI < 12 months, and de novo metastatic disease. *PFS* progression-free survival, *OS* overall survival, *ET* endocrine therapy, *TFI* treatment-free interval

Visceral metastases: Median rwPFS (95% CI) of patients without or with visceral metastasis was 28.3 months (21.7-34.9)/24.1 months (16.8-32.7) for 1L and 16.7 months (11.7-22.2)/ 14.3 months (9.6-19.0) for 2L treatment groups, respectively (Fig. 3A). Median OS (95% CI) of patients without or with visceral metastasis was NR (63.2-NE)/65.0 months (56.3-NE) for 1L and 50.9 months (43.2-NE)/48.7 months (38.8-57.2) for 2L treatment groups, respectively (Fig. 3B).

Liver metastases: Median rwPFS (95% CI) of patients without or with liver metastasis was 28.8 months (22.7-34.9)/12.8 months (8.3-24.1) for 1L and 17.8 months (14.4-22.8)/9.4 months (7.2-13.1) for 2L treatment groups, respectively (Fig. 3C). Median OS (95% CI) of patients without or with liver metastasis was NR (63.2-NE)/46.4 months (37.2-NE) for 1L and 54.1 months (49.0-65.1)/40.5 months (27.2-51.4) for 2L treatment groups, respectively (Fig. 3D).

Bone-only disease: Median OS (95% CI) was NR (57.8-NE) in 1L and 46.3 months (32.5-65.4) in 2L for patients with bone-only disease (Fig. 3E).

Treatment-free interval: Median OS (95% CI) for patients in the 1L treatment group with TFI ≥ 12 months and de novo metastasis was NR (NE-NE) and NR (56.3-NE), respectively; whereas for patients with TFI < 12 months median OS (95% CI) was 56.3 months (43.9-68.2) (Fig. 3F). Survival rates at 48 months were 72.9% (TFI ≥ 12 months), 70.9% (de novo metastasis), and 54.6% (TFI < 12 months), respectively.

### Analyses of patients initiating palbociclib at 125 mg/day

Baseline disease characteristics of the patients who initiated palbociclib at 125 mg/day and outcomes such as rwPFS and OS are shown in Supplemental Tables and Figures. The patient backgrounds, rwPFS, and OS were similar between the overall population and patients who initiated palbociclib treatment at 125 mg/day.

## Discussion

In this P-BRIDGE study, real-world, multi-center, observational study conducted in nearly 700 patients with HR+/HER2− ABC in Japan, the median OS with palbociclib treatment was over 5 years in 1L treatment after 48-months follow-up; the respective 5-year survival rate was 56.5%. Recently, the PALOMA-2 study, one of the pivotal studies of palbociclib, reported the median OS of 53.9 months (95% CI: 49.8-60.8) [11]. Several patient characteristics were similar between the PALOMA-2 and P-BRIDGE (1L population) studies, such as the presence of visceral metastasis (48.2% and 50.2%). However, there were differences in ECOG status (0, 1: 63.1%, 15.7% and 57.9%, 40.1%, respectively) and pre/perimenopausal status (0% and 20.1%) and in TFI. Notably, 20% of patients in the PALOMA-2 study had a TFI of ≤ 12 months, whereas 40% of patients in the P-BRIDGE study had a TFI of ≤ 12 months, indicating a different patient population in P-BRIDGE. Additionally, in P-BRIDGE, the combination of palbociclib with fulvestrant was the most frequently selected therapy. This choice may reflect its use in treating patients with a TFI < 12 months. This finding contrasts with the PALOMA-2 study, which was designed to examine letrozole as the sole hormone therapy combined with palbociclib. Another recent study, PARSIFAL-LONG [16], reported a median OS of 65.4 months (95% CI: 57.8-72.0) in the 5-year extended follow-up of PARSIFAL [17]. The main difference between the PARSIFAL-LONG and P-BRIDGE studies was the TFI population; PARSIFAL-LONG did not include patients with a TFI of < 12 months, focusing only on hormone-sensitive patients (ie. TFI > 12 months or de novo). In the P-BRIDGE study, the comparative population for hormone sensitivity included patients with a TFI ≥ 12 months and those with de novo metastatic disease. Although the median OS was not reached in our study, the 60-month survival rates were 72.9% (TFI ≥ 12 months) and 60% (de novo stage IV). Despite including 40% of patients with a TFI of ≤ 12 months, the median OS in the P-BRIDGE study exceeded five years, comparable to the PARSIFAL-LONG study.

Several studies have demonstrated the real-world effectiveness of palbociclib in clinical practice. In the P-REALITY X study [14], the real-world study in routine US clinical practice to date, the median PFS and OS were 19.8 months (95% CI, 17.9-21.7) and 49.1 months (95% CI, 45.2-57.7), respectively, in patients receiving first-line palbociclib (n = 1,572). The 2-year OS rate was 76.6%. When comparing patient backgrounds between P-BRIDGE and P-REALITY X, patients in P-BRIDGE tended to be younger (median age: 60 versus 70 years) and had a better ECOG Performance Status (0-1: 80% and 53%). More patients in P-BRIDGE had visceral (54% and 34%) and de novo metastatic disease at diagnosis (54% and 34%). Similarly, the PALBOSPAIN study, a retrospective, multi-center, observational, study evaluating real-world patterns and outcomes with first-line palbociclib in men and women with advanced HR+/HER2– breast cancer in Spain, reported a median real-world PFS and OS of 24 and 42 months, respectively [18]. The patient backgrounds were similar between P-BRIDGE and PALBOSPAIN, including frequencies of premenopausal patients (20.1% versus 15.6%), visceral metastasis (50.2% versus 54.9%), and TFI ≤ 12 months (45.8% versus 31.8%). Acknowledging caution in any comparison of rwPFS across different real-world studies, the respective median values in P-BRIDGE and PALBOSPAIN were similar (26.2 months [95% CI, 21.4-30.4] and 24 months [95% CI, 21-27]). However, median OS was longer in the P-BRIDGE study (68.2 months [95% CI, 60.8-NE]) compared to the PALBOSPAIN study (42 months [95% CI, 40-NE]), suggesting a potential contribution of differences in subsequent treatments between these two studies.

In the P-BRIDGE study, the proportion of subsequent therapies after palbociclib plus ET was similar to the PALOMA-2 study, including endocrine-based therapy (61.7% in the P-BRIDGE study and 60.8% in PALOMA-2 study, respectively) and chemotherapy (34.2% and 36.6%, respectively). However, 24.4% of patients in the P-BRIDGE study received CDK4/6 inhibitors plus ET as subsequent therapy after palbociclib plus ET, which differs from PALOMA-2 (0%). Positive results have recently been presented regarding post-CDK4/6 inhibitor treatments, including postMONARCH and CAPItello-291 [19, 20]. Such new treatment strategies might have impacted the OS outcome, underlining the requirement to further consider treatment strategies, such as combination therapy with molecular target drugs, to improve patients' lives.

Interestingly, a Japanese phase 2 study demonstrated a median OS of 85.4 months with a median follow-up of 89.7 months, investigating the efficacy and safety of first-line palbociclib combined with letrozole in postmenopausal Japanese women with ER+/HER2− ABC [21]. This phase 2 follow-up study also indicated a numerically longer median OS, and the reasons for this extended OS are being investigated, including factors such as subsequent therapy and other related aspects. Further, factors unique to Japan, such as subsequent treatment patterns, the medical environment, and the insurance system, might contribute to this OS result. The reason for the median OS over 5 years requires further investigation with RWD from Japan and other countries.

In the 1L treatment setting, patients without liver metastasis had longer OS compared with patients with liver metastasis (NR [68.0-NE] months and 46.4 [37.2-NE] months, respectively). This trend was also observed in rwPFS. As shown in Supplementary Tables 3-6, we could not find any differences in patients’ backgrounds as well as treatment patterns and dose modifications between those with/without liver metastasis (Supplementary Tables 3-6). These findings suggest a poorer prognosis for 1L patients with liver metastases; therefore, it is crucial to carefully consider the strategy of subsequent therapy in this population.

In 2L treatment group, some of the patient backgrounds were similar between PALOMA-3 and P-BRIDGE study such as ECOG PS 0 (59.7% and 57.3%) and incidence of visceral metastases (59.4% and 59.9%). Although PALOMA-3 study included multiple treatment lines (1L 24.2%, 2L 38.0%, 3L 25.9%, and more than 3L 11.8%), the median PFS was 11.2 months and OS was 34.8 months. Of note, the median OS was 38.0 months among the patient with only one prior line of therapy for metastatic disease [12]. In the P-BRIDGE study, rwPFS was 14.9 months and OS was 50.7 months in the 2L treatment group after 48.1 months follow-up, confirming the effectiveness of palbociclib as 2L treatment in Japanese routine clinical practice.

This real-world study has several potential limitations. This study is a retrospective data retrieval from electronic medical records, which may include missing or erroneous data. Some subgroups analysed had small sample sizes, which may have influenced the results. Short duration of follow-up, and lack of a control arm cause limitations especially in the assessment of time-to-event outcomes. Moreover, this study did not collect data on adverse events, given the challenges of reliable and consistent safety reporting in a retrospective chart review. Therefore, conclusions regarding the safety of palbociclib in the Japanese real-world setting cannot be directly drawn from this study. Disease progression was not based on standard criteria (e.g., Response Evaluation Criteria in Solid Tumors), but instead on the individual treating physician’s clinical assessment and interpretation of radiographic or pathologic results. Findings presented here may not be generalizable to patient populations in other country. Nevertheless, real-world data may be more representative of the effectiveness of treatment in the general population, and our study extends upon our understanding of outcomes associated with palbociclib combined with ET in Japanese patients.

In conclusion, the findings from this P-BRIDGE study derived from routine clinical practice in Japan further underline the clinical benefits observed thus far with palbociclib plus ET in the real-world setting. More than 5 years of median OS was observed in the 1L palbociclib plus ET treatment group, supporting the use of palbociclib plus ET as a 1L standard of care for HR+/HER2– ABC.

## Supplementary Information

Below is the link to the electronic supplementary material.

Supplementary file1 (PDF 1238 KB)

## Data Availability

Upon request, and subject to review, Pfizer will provide the data that support the findings of this study. Subject to certain criteria, conditions and exceptions, Pfizer may also provide access to the related individual de-identified participant data. See https://www.pfizer.com/science/clinical-trials/trial-data-and-results. for more information.
