# Correction: Overall survival of palbociclib plus endocrine therapy in Japanese patients with HR+/HER2− advanced breast cancer in the first-or second-line setting: a multicenter observational study (P-BRIDGE study)

**DOI:** 10.1007/s12282-025-01787-3

**Published:** 2026-01-27

**Authors:** Shigenori E. Nagai, Masaya Hattori, Tetsuhiro Yoshinami, Hiroko Masuda, Takuho Okamura, Kenichi Watanabe, Takahiro Nakayama, Michiko Tsuneizumi, Daisuke Takabatake, Michiko Harao, Hiroshi Yoshino, Natsuko Mori, Hiroyuki Yasojima, Chiya Oshiro, Madoka Iwase, Miki Yamaguchi, Takafumi Sangai, Shinsuke Sasada, Takanori Ishida, Manabu Futamura, Yasuaki Muramatsu, Nobuyoshi Kosaka, Norikazu Masuda

**Affiliations:** 1 Division of Breast Oncology, Saitama Prefectural Cancer Center, Saitama, Japan; 2 Department of Breast Oncology, Aichi Cancer Center, Nagoya, Japan; 3 Department of Breast and Endocrine Surgery, Graduate School of Medicine, Osaka University, Osaka, Japan; 4 Department of Breast Surgical Oncology, School of Medicine, Showa University, Tokyo, Japan; 5 Department of Breast Oncology, Tokai University School of Medicine, Tokyo, Japan; 6 Department of Breast Surgery, National Hospital Organization Hokkaido Cancer Center, Sapporo, Japan; 7 Department of Breast and Endocrine Surgery, Osaka International Cancer Institute, Osaka, Japan; 8 Department of Breast Surgery, Shizuoka General Hospital, Shizuoka, Japan; 9 Department of Breast Oncology, National Hospital Organization Shikoku Cancer Center, Matsuyama, Japan; 10 Department of Breast Oncology, Jichi Medical University, Shimotsuke, Japan; 11 Breast and Endocrinological Surgery, Ishikawa Prefectural Central Hospital, Kanazawa, Japan; 12 Department of Breast Surgery, Seirei Hamamatsu General Hospital, Hamamatsu, Japan; 13 Department of Surgery, Breast Oncology, National Hospital Organization Osaka National Hospital, Osaka, Japan; 14 Department of Breast Surgery, Kaizuka City Hospital, Osaka, Japan; 15 Department of Breast and Endocrine Surgery, Nagoya University Hospital, Nagoya, Japan; 16 Department of Breast Surgery, JCHO Kurume General Hospital, Kurume, Japan; 17 Department of Breast and Thyroid Surgery, Kitasato University School of Medicine, Sagamihara, Japan; 18 Department of Surgical Oncology, Research Institute for Radiation Biology and Medicine, Hiroshima University, Hiroshima, Japan; 19 Division of Breast and Endocrine Surgical Oncology, Tohoku University Graduate School of Medicine, Sendai, Japan; 20 Department of Breast Surgery, Gifu University Hospital, Gifu, Japan; 21 Oncology Medical Affairs, Pfizer Japan Inc, Tokyo, Japan; 22 Department of Breast Surgery, Graduate School of Medicine, Kyoto University, 54 Shogoin-Kawahara-Cho, Sakyo-Ku, Kyoto, 606-8507 Japan

**Correction: Breast Cancer (2025) 32:705–715** 10.1007/s12282-025-01689-4

The sentence beginning “Bone-only disease: Median OS …” in this article should have read “Bone-only disease: Median OS (95% CI) was NR (57.8–NE) in 1L and 49.9 months (35.8–NE) in 2L for patients with bone-only disease (Fig. 3E).”

Figure 3E and Supplementary Fig. 2C in the original version of this article have been replaced to show the OS data for the bone metastasis subgroup with the data measured from the initiation of palbociclib.

**Figure 3E**

**Incorrect version:** 
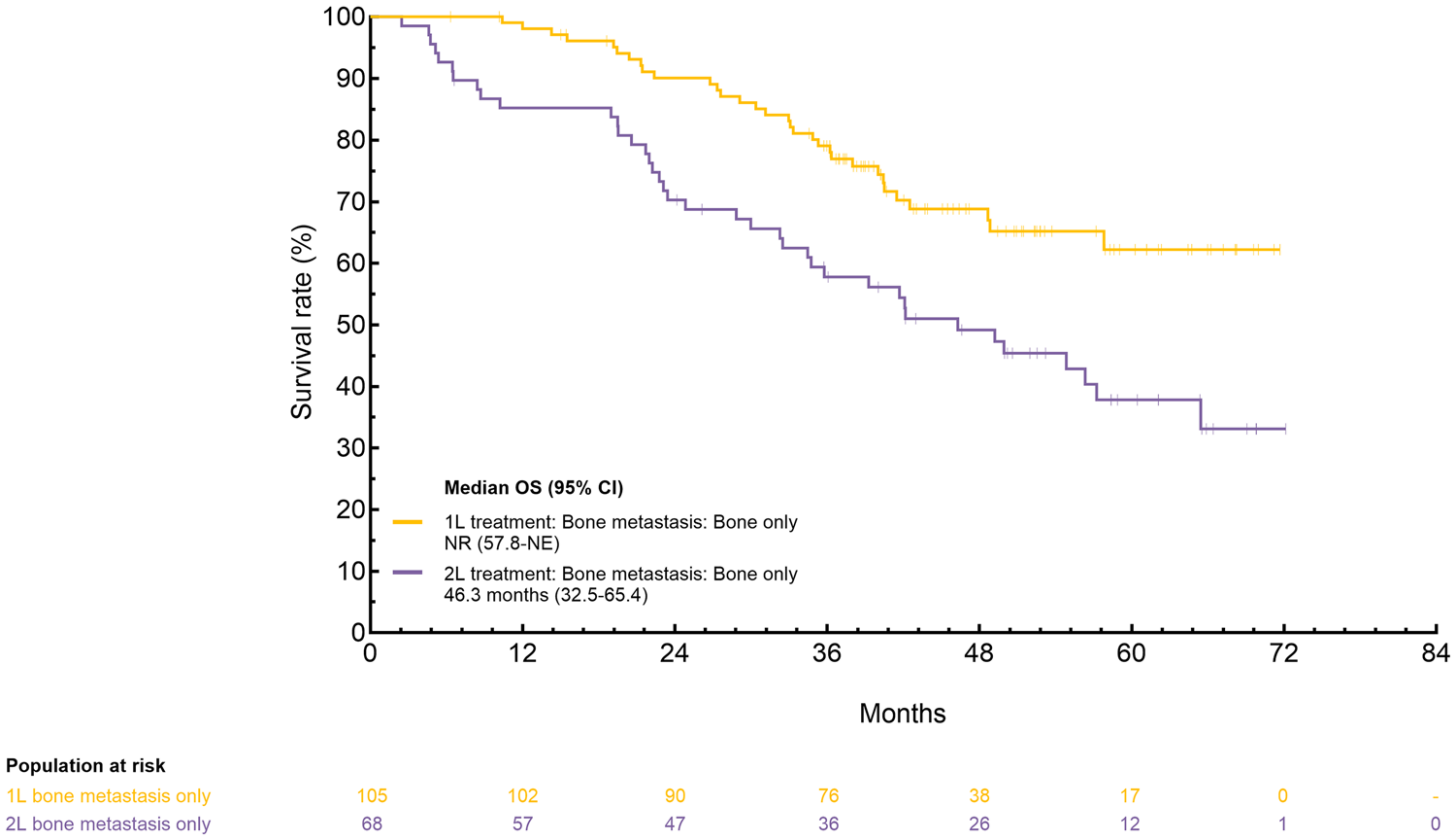


**Corrected version:** 
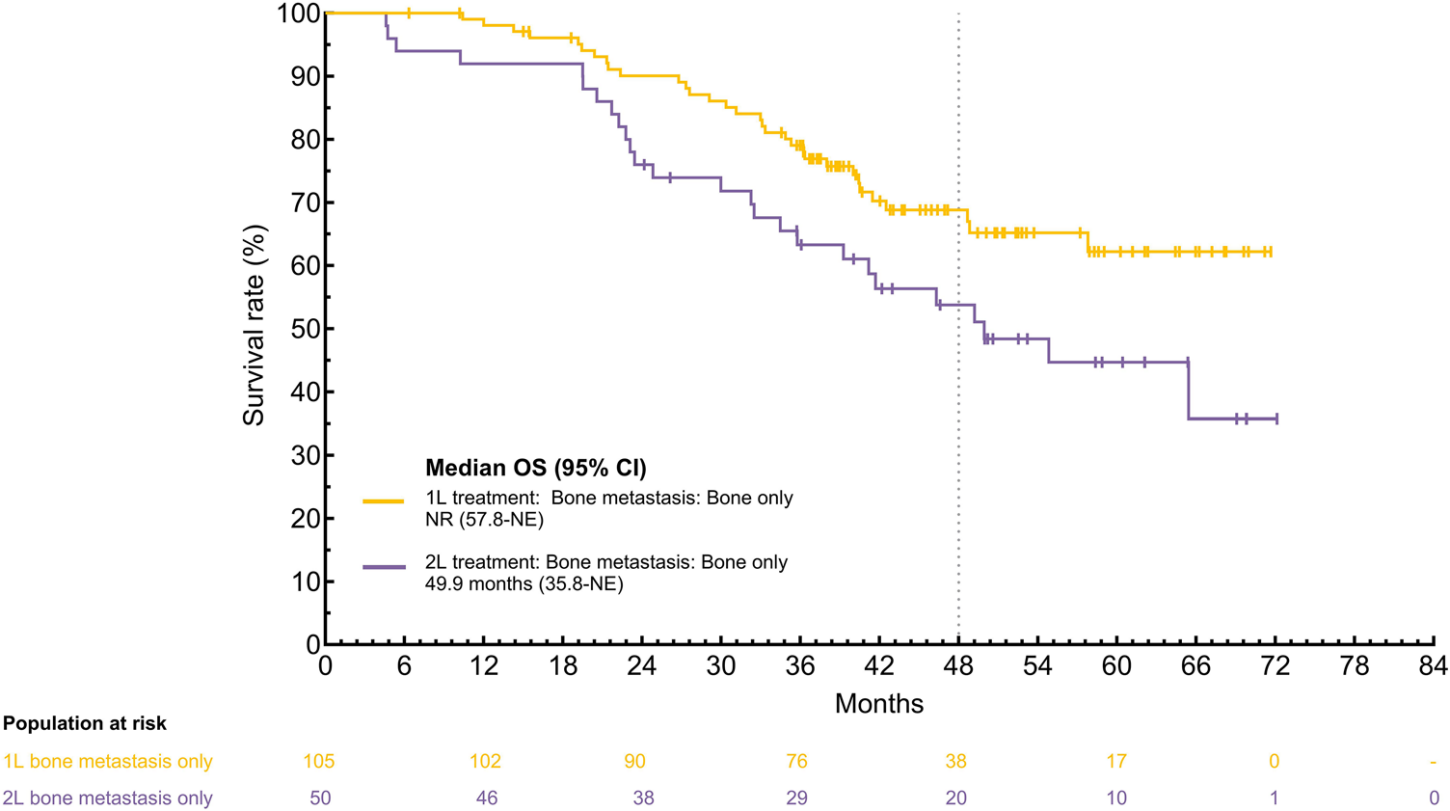


**Supplementary Fig. 1**

**Incorrect version:**

**Supplementary Fig. 1**

Real-world A) PFS B) OS and C) CFS of palbociclib plus ET as 1L and 2L treatment in patients with ABC who started palbociclib 125 mg/day. 1L, first-line; 2L, second-line; ABC, advanced breast cancer; CFS, chemotherapy-free survival; CI, confidence interval; ET, endocrine therapy; NE, not evaluated; OS, overall survival; PFS, progression-free survival

**Corrected version:**

**Supplementary Fig. 1**

Real-world A) PFS B) OS and C) CFS of palbociclib plus ET as 1L and 2L treatment in patients with ABC who started palbociclib 125 mg/day. 1L, first-line; 2L, second-line; ABC, advanced breast cancer; CFS, chemotherapy-free survival; CI, confidence interval; ET, endocrine therapy; NE, not estimated; OS, overall survival; PFS, progression-free survival

**Supplementary Fig. 2**

**Incorrect version:**

**Supplementary Fig. 2** Subgroup analysis of OS with palbociclib plus ET as 1L and 2L treatment in patients with ABC stratified by A) presence/absence of visceral metastasis B) presence/absence of liver metastasis C) presence/absence of bone only metastasis and D) treatment-free interval.1L, first-line; 2L, second-line; ABC, advanced breast cancer; CI, confidence interval; ET, endocrine therapy; NE, not evaluated; OS, overall survival

**Corrected version:**

**Supplementary Fig. 2** Subgroup analysis of OS with palbociclib plus ET as 1L and 2L treatment in patients with ABC who started palbociclib 125 mg/day stratified by A) presence/absence of visceral metastasis B) presence/absence of liver metastasis C) presence/absence of bone only metastasis and D) treatment-free interval.1L, first-line; 2L, second-line; ABC, advanced breast cancer; CI, confidence interval; ET, endocrine therapy; NE, not estimated; OS, overall survival

**Supplementary Fig. 2C**

**Incorrect version:** 
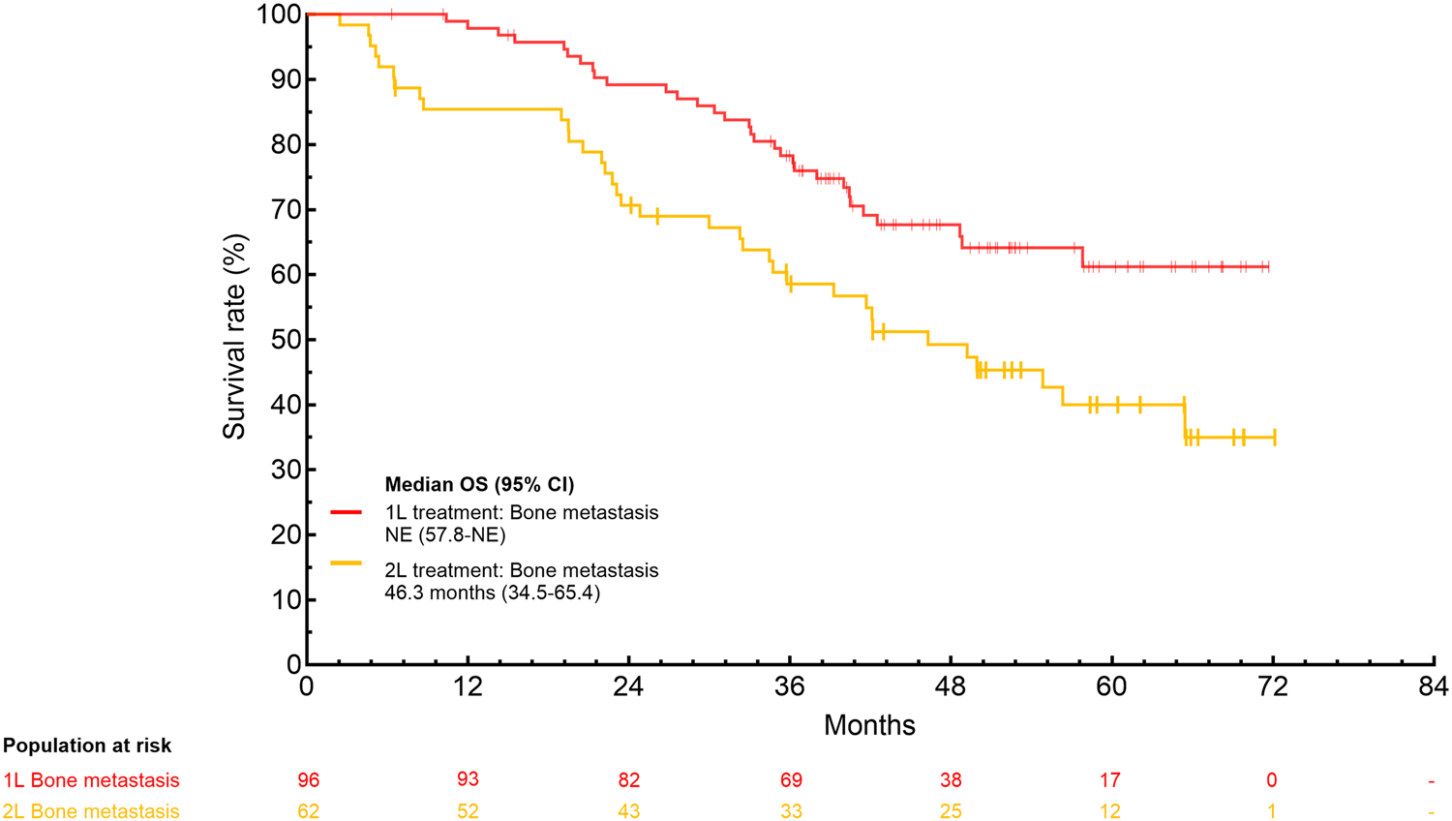


**Corrected version:** 
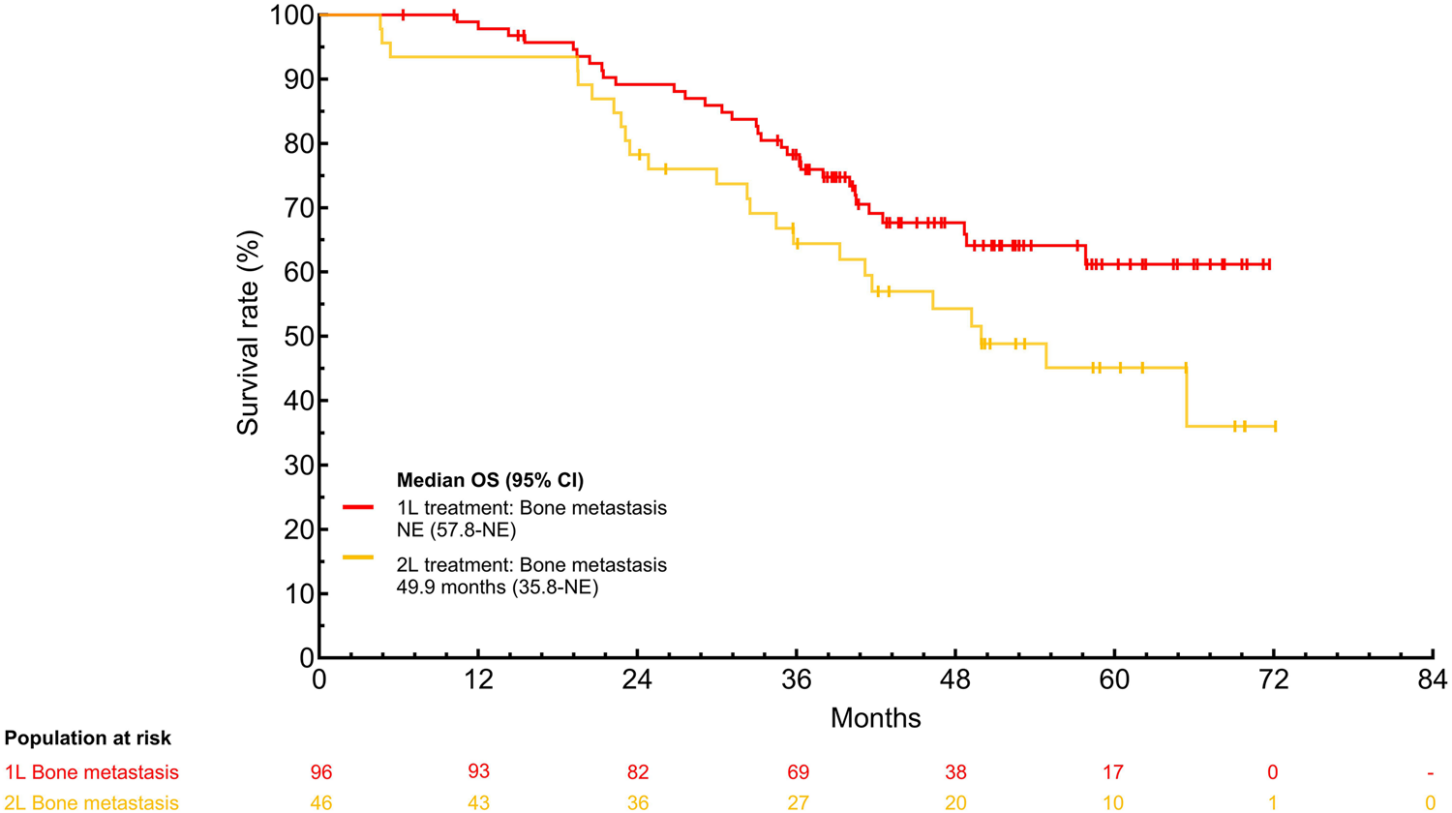


In Supplementary Tables 1, 3, and 5, for each patient background subgroup—including the group starting at 125 mg, the subgroups with or without visceral metastasis, and the subgroups with or without of liver metastasis—the numbers of patients with a treatment free interval (TFI) < 12 months and ≥ 12 months were inadvertently listed in reverse order. Supplementary Tables 1, 3, and 5 have now been corrected.

**Supplementary appendix**

**Supplementary Table 1** Demographics and clinical characteristics of patients with ABC who started palbociclib 125 mg/day

**Incorrect version:**

**Table:** 

	1L (n = 385)	2L (n = 233)
Age (years), median (range)	59.0 (29.0, 85.0)	60.0 (32.0, 87.0)
Age category (years), n (%)		
≤ 49	86 (22.3)	52 (22.3)
50–64	168 (43.6)	91 (39.1)
65–74	104 (27.0)	58 (24.9)
≥ 75	27 (7.0)	32 (13.7)
Sex, n (%)		
Male	2 (0.5)	1 (0.4)
Female	383 (99.5)	232 (99.6)
Menopausal status, n (%)^a^		
Premenopausal/perimenopausal	84 (21.9)	61 (26.3)
Postmenopausal	265 (69.2)	155 (66.8)
Unknown	34 (8.9)	16 (6.9)
ECOG PS		
0	241 (62.6)	136 (58.4)
1	60 (15.6)	53 (22.7)
2	7 (1.8)	0
3	5 (1.3)	1 (0.4)
4	0	1 (0.4)
Unknown	72 (18.7)	42 (18.0)
Disease sites, n (%)		
Visceral metastasis	192 (49.9)	140 (60.1)
Liver metastasis	64 (16.6)	65 (27.9)
Bone-only metastasis	96 (24.9)	46 (19.7)
DFI, n (%)^b^		
≥ 24 months	253 (65.7)	146 (62.7)
< 24 months	34 (8.8)	18 (7.7)
TFI, n (%)^c^		
De novo metastasis/others	100 (26.0)	64 (27.5)
≥ 12 months	174 (45.2)	93 (39.9)
< 12 months	80 (20.8)	44 (18.9)
Symptoms at the start of palbociclib, n (%)^d^		
Yes	203 (52.7)	83 (35.6)
No	166 (43.1)	140 (60.1)
Unknown	16 (4.2)	10 (4.3)
Prior ET for (neo)adjuvant, n (%)	266 (69.1)	155 (66.5)
Prior CT for (neo)adjuvant, n (%)	199 (51.7)	111 (47.6)
Induction CT, n (%)	21 (5.5)	8 (3.4)

ABC: advanced breast cancer, CT: chemotherapy, DFI: disease-free interval (the time from the date of breast cancer surgery to the diagnosis date of recurrence), ECOG PS: Eastern Cooperative Oncology Group performance status, ET: endocrine therapy, TFI: treatment-free interval (the time from the end of adjuvant therapy to the diagnosis date of recurrence)

^a^The denominator is the number of female patients

^b^Percentage was calculated based on patients with disease stage other than “stage IV”. The patients without the date of breast cancer surgery were excluded from this calculation

^c^“Others” included patients who had surgery but did not undergo adjuvant therapy. The patients without the date of breast cancer surgery were excluded from this calculation

^d^Symptoms included bone pain, shortness of breath, coughing, headaches, dizziness, nausea, swelling around the neck and armpits, numbness in the limbs, abdominal bloating, and jaundice

**Corrected version:**

**Table:** 

	1L (n = 385)	2L (n = 233)
Age (years), median (range)	59.0 (29.0, 85.0)	60.0 (32.0, 87.0)
Age category (years), n (%)		
≤ 49	86 (22.3)	52 (22.3)
50–64	168 (43.6)	91 (39.1)
65–74	104 (27.0)	58 (24.9)
≥ 75	27 (7.0)	32 (13.7)
Sex, n (%)		
Male	2 (0.5)	1 (0.4)
Female	383 (99.5)	232 (99.6)
Menopausal status, n (%)^a^		
Premenopausal/perimenopausal	84 (21.9)	61 (26.3)
Postmenopausal	265 (69.2)	155 (66.8)
Unknown	34 (8.9)	16 (6.9)
ECOG PS		
0	241 (62.6)	136 (58.4)
1	60 (15.6)	53 (22.7)
2	7 (1.8)	0
3	5 (1.3)	1 (0.4)
4	0	1 (0.4)
Unknown	72 (18.7)	42 (18.0)
Disease sites, n (%)		
Visceral metastasis	192 (49.9)	140 (60.1)
Liver metastasis	64 (16.6)	65 (27.9)
Bone-only metastasis	96 (24.9)	46 (19.7)
DFI, n (%)^b^		
≥ 24 months	253 (65.7)	146 (62.7)
< 24 months	34 (8.8)	18 (7.7)
TFI, n (%)^c^		
De novo metastasis/others	100 (26.0)	64 (27.5)
≥ 12 months	80 (20.8)	44 (18.9)
< 12 months	174 (45.2)	93 (39.9)
Symptoms at the start of palbociclib, n (%)^d^		
Yes	203 (52.7)	83 (35.6)
No	166 (43.1)	140 (60.1)
Unknown	16 (4.2)	10 (4.3)
Prior ET for (neo)adjuvant, n (%)	266 (69.1)	155 (66.5)
Prior CT for (neo)adjuvant, n (%)	199 (51.7)	111 (47.6)
Induction CT, n (%)	21 (5.5)	8 (3.4)

ABC: advanced breast cancer, CT: chemotherapy, DFI: disease-free interval (the time from the date of breast cancer surgery to the diagnosis date of recurrence), ECOG PS: Eastern Cooperative Oncology Group performance status, ET: endocrine therapy, TFI: treatment-free interval (the time from the end of adjuvant therapy to the diagnosis date of recurrence)

^a^The denominator is the number of female patients

^b^Percentage was calculated based on patients with disease stage other than “stage IV”. The patients without the date of breast cancer surgery were excluded from this calculation

^c^“Others” included patients who had surgery but did not undergo adjuvant therapy. The patients without the date of breast cancer surgery were excluded from this calculation

^d^Symptoms included bone pain, shortness of breath, coughing, headaches, dizziness, nausea, swelling around the neck and armpits, numbness in the limbs, abdominal bloating, and jaundice

**Supplementary Table 3** Demographics and clinical characteristics of patients’ presence/absence of visceral metastasis

**Incorrect version:**

**Table:** 

	1L		2L	
	absence of visceral metastasis (n = 212)	presence of visceral metastasis (n = 214)	absence of visceral metastasis (n = 107)	presence of visceral metastasis (n = 160)
Age category (years), n (%)				
≤ 49	37 (17.5)	51 (23.8)	28 (26.2)	32 (20.0)
50–64	101 (47.6)	77 (36.0)	43 (40.2)	58 (36.3)
65–74	56 (26.4)	62 (29.0)	25 (23.4)	41 (25.6)
≥ 75	18 (8.5)	24 (11.2)	11 (10.3)	29 (18.1)
Sex, n (%)				
Male	1 (0.5)	2 (0.9)	1 (0.9)	0
Female	211 (99.5)	212 (99.1)	106 (99.1)	160 (100.0)
Menopausal status, n (%)^a^				
Pre/perimenopausal	38 (18.0)	47 (22.2)	31 (29.2)	38 (23.8)
Postmenopausal	155 (73.5)	147 (69.3)	66 (62.3)	114 (71.3)
Unknown	18 (8.5)	18 (8.5)	9 (8.5)	8 (5.0)
ECOG PS, n (%)				
0	135 (63.7)	134 (62.6)	52 (48.6)	101 (63.1)
1	30 (14.2)	37 (17.3)	38 (35.5)	25 (15.6)
≥ 2	5 (2.4)	7 (3.3)	1 (0.9)	2 (1.3)
Unknown	42 (19.8)	36 (16.8)	16 (15.0)	32 (20.0)
Disease sites, n (%)				
Visceral metastasis	212 (100.0)	214 (100.0)	107 (100.0)	160 (100.0)
Liver metastasis	0	72 (33.6)	0	73 (45.6)
Bone-only metastasis	105 (49.5)	0	50 (46.7)	0
DFI, n (%)^b^				
< 24 months	21 (9.9)	18 (8.4)	9 (8.4)	14 (8.8)
≥ 24 months	140 (66.0)	138 (64.5)	68 (63.6)	97 (60.6)
TFI, n (%)^c^				
De novo metastasis/others	55 (25.9)	56 (26.2)	27 (25.2)	48 (30.0)
≥ 12 months	102 (48.1)	93 (43.5)	46 (43.0)	59 (36.9)
< 12 months	41 (19.3)	46 (21.5)	20 (18.7)	34 (21.3)
Symptoms at the start of palbociclib, n (%)^d^				
Yes	94 (44.3)	97 (45.3)	68 (63.6)	95 (59.4)
No	110 (51.9)	107 (50.0)	36 (33.6)	56 (35.0)
Unknown	8 (3.8)	10 (4.7)	3 (2.8)	9 (5.6)
Prior ET for (neo)adjuvant, n (%)	142 (67.0)	149 (69.6)	71 (66.4)	105 (65.6)
Prior CT for (neo)adjuvant, n (%)	117 (55.2)	100 (46.7)	57 (53.3)	68 (42.5)

CT: chemotherapy, DFI: disease-free interval (the time from the date of breast cancer surgery to the diagnosis date of recurrence), ECOG PS: Eastern Cooperative Oncology Group performance status, ET: endocrine therapy, TFI: treatment-free interval (the time from the end of adjuvant therapy to the diagnosis date of recurrence)

^a^The denominator is the number of female patients

^b^Percentage was calculated based on patients with disease stage other than “stage IV”. The patients without the date of breast cancer surgery were excluded from this calculation

^c^“Others” included patients who had surgery but did not undergo adjuvant therapy. The patients without the date of breast cancer surgery were excluded from this calculation

^d^Symptoms included bone pain, shortness of breath, coughing, headaches, dizziness, nausea, swelling around the neck and armpits, numbness in the limbs, abdominal bloating, and jaundice

**Corrected version:**

**Table:** 

	1L		2L	
	absence of visceral metastasis (n = 212)	presence of visceral metastasis (n = 214)	absence of visceral metastasis (n = 107)	presence of visceral metastasis (n = 160)
Age category (years), n (%)				
≤ 49	37 (17.5)	51 (23.8)	28 (26.2)	32 (20.0)
50–64	101 (47.6)	77 (36.0)	43 (40.2)	58 (36.3)
65–74	56 (26.4)	62 (29.0)	25 (23.4)	41 (25.6)
≥ 75	18 (8.5)	24 (11.2)	11 (10.3)	29 (18.1)
Sex, n (%)				
Male	1 (0.5)	2 (0.9)	1 (0.9)	0
Female	211 (99.5)	212 (99.1)	106 (99.1)	160 (100.0)
Menopausal status, n (%)^a^				
Pre/perimenopausal	38 (18.0)	47 (22.2)	31 (29.2)	38 (23.8)
Postmenopausal	155 (73.5)	147 (69.3)	66 (62.3)	114 (71.3)
Unknown	18 (8.5)	18 (8.5)	9 (8.5)	8 (5.0)
ECOG PS, n (%)				
0	135 (63.7)	134 (62.6)	52 (48.6)	101 (63.1)
1	30 (14.2)	37 (17.3)	38 (35.5)	25 (15.6)
≥ 2	5 (2.4)	7 (3.3)	1 (0.9)	2 (1.3)
Unknown	42 (19.8)	36 (16.8)	16 (15.0)	32 (20.0)
Disease sites, n (%)				
Visceral metastasis	212 (100.0)	214 (100.0)	107 (100.0)	160 (100.0)
Liver metastasis	0	72 (33.6)	0	73 (45.6)
Bone-only metastasis	105 (49.5)	0	50 (46.7)	0
DFI, n (%)^b^				
< 24 months	21 (9.9)	18 (8.4)	9 (8.4)	14 (8.8)
≥ 24 months	140 (66.0)	138 (64.5)	68 (63.6)	97 (60.6)
TFI, n (%)^c^				
De novo metastasis/others	55 (25.9)	56 (26.2)	27 (25.2)	48 (30.0)
≥ 12 months	41 (19.3)	46 (21.5)	20 (18.7)	34 (21.3)
< 12 months	102 (48.1)	93 (43.5)	46 (43.0)	59 (36.9)
Symptoms at the start of palbociclib, n (%)^d^				
Yes	94 (44.3)	97 (45.3)	68 (63.6)	95 (59.4)
No	110 (51.9)	107 (50.0)	36 (33.6)	56 (35.0)
Unknown	8 (3.8)	10 (4.7)	3 (2.8)	9 (5.6)
Prior ET for (neo)adjuvant, n (%)	142 (67.0)	149 (69.6)	71 (66.4)	105 (65.6)
Prior CT for (neo)adjuvant, n (%)	117 (55.2)	100 (46.7)	57 (53.3)	68 (42.5)

CT: chemotherapy, DFI: disease-free interval (the time from the date of breast cancer surgery to the diagnosis date of recurrence), ECOG PS: Eastern Cooperative Oncology Group performance status, ET: endocrine therapy, TFI: treatment-free interval (the time from the end of adjuvant therapy to the diagnosis date of recurrence)

^a^The denominator is the number of female patients

^b^Percentage was calculated based on patients with disease stage other than “stage IV”. The patients without the date of breast cancer surgery were excluded from this calculation

^c^“Others” included patients who had surgery but did not undergo adjuvant therapy. The patients without the date of breast cancer surgery were excluded from this calculation

^d^Symptoms included bone pain, shortness of breath, coughing, headaches, dizziness, nausea, swelling around the neck and armpits, numbness in the limbs, abdominal bloating, and jaundice

**Supplementary Table 5** Demographics and clinical characteristics of patients’ presence/absence of liver metastasis

**Incorrect version:**

**Table:** 

	1L		2L	
	absence of liver metastasis (n = 354)	presence of liver metastasis (n = 72)	absence of liver metastasis (n = 194)	presence of liver metastasis (n = 73)
Age category (years), n (%)				
≤ 49	63 (17.8)	25 (34.7)	46 (23.7)	14 (19.2)
50–64	152 (42.9)	26 (36.1)	71 (36.6)	30 (41.1)
65–74	101 (28.5)	17 (23.6)	48 (24.7)	18 (24.7)
≥ 75	38 (10.7)	4 (5.6)	29 (14.9)	11 (15.1)
Sex, n (%)				
Male	3 (0.8)	0	1 (0.5)	0
Female	351 (99.2)	72 (100.0)	193 (99.5)	73 (100.0)
Menopausal status, n (%)^a^				
Pre/perimenopausal	65 (18.5)	20 (27.8)	51 (26.4)	18 (24.7)
Postmenopausal	253 (72.1)	49 (68.1)	129 (66.8)	51 (69.9)
Unknown	33 (9.4)	3 (4.2)	13 (6.7)	4 (5.5)
ECOG PS, n (%)				
0	222 (62.7)	47 (65.3)	108 (55.7)	45 (61.6)
1	52 (14.7)	15 (20.8)	54 (27.8)	9 (12.3)
≥ 2	10 (2.8)	2 (2.8)	3 (1.5)	0
Unknown	70 (19.8)	8 (11.1)	29 (14.9)	19 (26.0)
Disease sites, n (%)				
Visceral metastasis	142 (40.1)	72 (100.0)	87 (44.8)	73 (100.0)
Liver metastasis	0	72 (100.0)	0	73 (100.0)
Bone-only metastasis	105 (29.7)	0	50 (25.8)	0
DFI, n (%)^b^				
< 24 months	28 (7.9)	11 (15.3)	13 (6.7)	10 (13.7)
≥ 24 months	233 (65.8)	45 (62.5)	122 (62.9)	43 (58.9)
TFI, n (%)^c^				
De novo metastasis/others	95 (26.8)	16 (22.2)	56 (28.9)	19 (26.0)
≥ 12 months	154 (43.5)	41 (56.9)	69 (35.6)	36 (49.3)
< 12 months	74 (20.9)	13 (18.1)	44 (22.7)	10 (13.7)
Symptoms at the start of palbociclib, n (%)^d^				
Yes	152 (42.9)	39 (54.2)	117 (60.3)	46 (63.0)
No	188 (53.1)	29 (40.3)	69 (35.6)	23 (31.5)
Unknown	14 (4.0)	4 (5.6)	8 (4.1)	4 (5.5)
Prior ET for (neo)adjuvant, n (%)	236 (66.7)	55 (76.4)	124 (63.9)	52 (71.2)
Prior CT for (neo)adjuvant, n (%)	176 (49.7)	41 (56.9)	90 (46.4)	35 (47.9)

CT: chemotherapy, DFI: disease-free interval (the time from the date of breast cancer surgery to the diagnosis date of recurrence), ECOG PS: Eastern Cooperative Oncology Group performance status, ET: endocrine therapy, TFI: treatment-free interval (the time from the end of adjuvant therapy to the diagnosis date of recurrence)

^a^The denominator is the number of female patients

^b^Percentage was calculated based on patients with disease stage other than “stage IV”. The patients without the date of breast cancer surgery were excluded from this calculation

^c^“Others” included patients who had surgery but did not undergo adjuvant therapy. The patients without the date of breast cancer surgery were excluded from this calculation

^d^Symptoms included bone pain, shortness of breath, coughing, headaches, dizziness, nausea, swelling around the neck and armpits, numbness in the limbs, abdominal bloating, and jaundice

**Corrected version:**

**Table:** 

	1L		2L	
	absence of liver metastasis (n = 354)	presence of liver metastasis (n = 72)	absence of liver metastasis (n = 194)	presence of liver metastasis (n = 73)
Age category (years), n (%)				
≤ 49	63 (17.8)	25 (34.7)	46 (23.7)	14 (19.2)
50–64	152 (42.9)	26 (36.1)	71 (36.6)	30 (41.1)
65–74	101 (28.5)	17 (23.6)	48 (24.7)	18 (24.7)
≥ 75	38 (10.7)	4 (5.6)	29 (14.9)	11 (15.1)
Sex, n (%)				
Male	3 (0.8)	0	1 (0.5)	0
Female	351 (99.2)	72 (100.0)	193 (99.5)	73 (100.0)
Menopausal status, n (%)^a^				
Pre/perimenopausal	65 (18.5)	20 (27.8)	51 (26.4)	18 (24.7)
Postmenopausal	253 (72.1)	49 (68.1)	129 (66.8)	51 (69.9)
Unknown	33 (9.4)	3 (4.2)	13 (6.7)	4 (5.5)
ECOG PS, n (%)				
0	222 (62.7)	47 (65.3)	108 (55.7)	45 (61.6)
1	52 (14.7)	15 (20.8)	54 (27.8)	9 (12.3)
≥ 2	10 (2.8)	2 (2.8)	3 (1.5)	0
Unknown	70 (19.8)	8 (11.1)	29 (14.9)	19 (26.0)
Disease sites, n (%)				
Visceral metastasis	142 (40.1)	72 (100.0)	87 (44.8)	73 (100.0)
Liver metastasis	0	72 (100.0)	0	73 (100.0)
Bone-only metastasis	105 (29.7)	0	50 (25.8)	0
DFI, n (%)^b^				
< 24 months	28 (7.9)	11 (15.3)	13 (6.7)	10 (13.7)
≥ 24 months	233 (65.8)	45 (62.5)	122 (62.9)	43 (58.9)
TFI, n (%)^c^				
De novo metastasis/others	95 (26.8)	16 (22.2)	56 (28.9)	19 (26.0)
≥ 12 months	74 (20.9)	13 (18.1)	44 (22.7)	10 (13.7)
< 12 months	154 (43.5)	41 (56.9)	69 (35.6)	36 (49.3)
Symptoms at the start of palbociclib, n (%)^d^				
Yes	152 (42.9)	39 (54.2)	117 (60.3)	46 (63.0)
No	188 (53.1)	29 (40.3)	69 (35.6)	23 (31.5)
Unknown	14 (4.0)	4 (5.6)	8 (4.1)	4 (5.5)
Prior ET for (neo)adjuvant, n (%)	236 (66.7)	55 (76.4)	124 (63.9)	52 (71.2)
Prior CT for (neo)adjuvant, n (%)	176 (49.7)	41 (56.9)	90 (46.4)	35 (47.9)

CT: chemotherapy, DFI: disease-free interval (the time from the date of breast cancer surgery to the diagnosis date of recurrence), ECOG PS: Eastern Cooperative Oncology Group performance status, ET: endocrine therapy, TFI: treatment-free interval (the time from the end of adjuvant therapy to the diagnosis date of recurrence)

^a^The denominator is the number of female patients

^b^Percentage was calculated based on patients with disease stage other than “stage IV”. The patients without the date of breast cancer surgery were excluded from this calculation

^c^“Others” included patients who had surgery but did not undergo adjuvant therapy. The patients without the date of breast cancer surgery were excluded from this calculation

^d^Symptoms included bone pain, shortness of breath, coughing, headaches, dizziness, nausea, swelling around the neck and armpits, numbness in the limbs, abdominal bloating, and jaundice

