# Supplementary material for: Overall survival of palbociclib plus endocrine therapy in Japanese patients with HR+/HER2– advanced breast cancer in the first-or second-line setting: a multicenter observational study (P-BRIDGE study)
Source: Breast Cancer. 2025 Apr 5;32(4):705–15. doi: 10.1007/s12282-025-01689-4 (PMC12174214; doi:10.1007/s12282-025-01689-4)
Supplement: Supplementary file 1 — Supplementary file1 (PDF 1238 KB) [file 12282_2025_1689_MOESM1_ESM.pdf]

Supplementary Appendix

Supplementary Table 1. Demographics and clinical characteristics of patients with ABC who started palbociclib 125 mg/day

	1L<br>(n=385)	2L<br>(n=233)
Age (years), median (range)	59.0 (29.0, 85.0)	60.0 (32.0, 87.0)
Age category (years), n (%)		
≤49	86 (22.3)	52 (22.3)
50–64	168 (43.6)	91 (39.1)
65–74	104 (27.0)	58 (24.9)
≥75	27 (7.0)	32 (13.7)
Sex, n (%)		
Male	2 (0.5)	1 (0.4)
Female	383 (99.5)	232 (99.6)
Menopausal status, n (%) [a]		
Premenopausal/perimenopausal	84 (21.9)	61 (26.3)
Postmenopausal	265 (69.2)	155 (66.8)
Unknown	34 (8.9)	16 (6.9)
ECOG PS		
0	241 (62.6)	136 (58.4)
1	60 (15.6)	53 (22.7)
2	7 (1.8)	0

3	5 (1.3)	1 (0.4)
4	0	1 (0.4)
Unknown	72 (18.7)	42 (18.0)
Disease sites, n (%)		
Visceral metastasis	192 (49.9)	140 (60.1)
Liver metastasis	64 (16.6)	65 (27.9)
Bone-only metastasis	96 (24.9)	46 (19.7)
DFI, n (%) [b]		
≥24 months	253 (65.7)	146 (62.7)
<24 months	34 (8.8)	18 (7.7)
TFI, n (%) [c]		
De novo metastasis/others	100 (26.0)	64 (27.5)
≥ 12 months	174 (45.2)	93 (39.9)
< 12 months	80 (20.8)	44 (18.9)
Symptoms at the start of palbociclib, n (%) [d]		
Yes	203 (52.7)	83 (35.6)
No	166 (43.1)	140 (60.1)
Unknown	16 (4.2)	10 (4.3)
Prior ET for (neo)adjuvant, n (%)	266 (69.1)	155 (66.5)
Prior CT for (neo)adjuvant, n (%)	199 (51.7)	111 (47.6)
Induction CT, n (%)	21 (5.5)	8 (3.4)

ABC : advanced breast cancer, CT : chemotherapy, DFI : disease-free interval (the time from the date of breast cancer surgery to the diagnosis date of recurrence), ECOG PS : Eastern Cooperative Oncology Group performance status, ET : endocrine therapy, TFI : treatment-free interval (the time from the end of adjuvant therapy to the diagnosis date of recurrence)
a The denominator is the number of female patients
b Percentage was calculated based on patients with disease stage other than “stage IV”. The patients without the date of breast cancer surgery were excluded from this calculation
c “Others” included patients who had surgery but did not undergo adjuvant therapy. The patients without the date of breast cancer surgery were excluded from this calculation
d Symptoms included bone pain, shortness of breath, coughing, headaches, dizziness, nausea, swelling around the neck and armpits, numbness in the limbs, abdominal bloating, and jaundice

Supplementary Table 2. Real-world treatment pattern and dose modification in patients with ABC who started palbociclib 125 mg/day

	1L treatment (n=385) n (%)	2L treatment (n=233) n (%)
Status of palbociclib administration [a]		
Ongoing	80 (20.8)	30 (12.9)
Discontinued	305 (79.2)	203 (87.1)
Reason for discontinuation of palbociclib[b, c]		
Disease progression	211 (69.2)	156 (76.8)
Adverse event	63 (20.7)	38 (18.7)
Other	37 (12.1)	14 (6.9)
Patients requiring dose reduction		
No	89 (23.1)	60 (25.8)
Yes	296 (76.9)	173 (74.2)
100 (mg/day)	106 (27.5)	70 (30.0)
75 (mg/day)	177 (46.0)	96 (41.2)
Other (mg¥day)	13 (3.4)	7 (3.0)
Timing of first dose reduction of palbociclib [d]		
≤3 months	231 (78.0)	139 (80.3)
3–≤6 months	31 (10.5)	14 (8.1)
6–≤9 months	10 (3.4)	6 (3.5)
>9 months	24 (8.1)	14 (8.1)
Endocrine therapy in combination with palbociclib[e]		
Fulvestrant	214 (55.6)	183 (78.5)
Letrozole	151 (39.2)	38 (16.3)
Anastrozole	15 (3.9)	9 (3.9)
Exemestane	2 (0.5)	2 (0.9)
Tamoxifen	5 (1.3)	2 (0.9)

[a]Data cutoff date was February 16, 2024.

[b]Percentage was calculated with patients who discontinued palbociclib.

[c]The different reasons for palbociclib discontinuation in the same patient were counted in the respective group.

[d]Percentage was calculated with patients who underwent dose reduction.

[e]The different endocrine therapies used in the same patient within the same treatment line were counted in the respective group.

Supplementary Table 3. Demographics and clinical characteristics of patients' presence/absence of visceral metastasis

	1L		2L	
	absence of visceral metastasis (n=212)	presence of visceral metastasis (n=214)	absence of visceral metastasis (n=107)	presence of visceral metastasis (n=160)
Age category (years), n (%)				
≤49	37 (17.5)	51 (23.8)	28 (26.2)	32 (20.0)
50–64	101 (47.6)	77 (36.0)	43 (40.2)	58 (36.3)
65–74	56 (26.4)	62 (29.0)	25 (23.4)	41 (25.6)
≥75	18 (8.5)	24 (11.2)	11 (10.3)	29 (18.1)
Sex, n (%)				
Male	1 (0.5)	2 (0.9)	1 (0.9)	0
Female	211 (99.5)	212 (99.1)	106 (99.1)	160 (100.0)
Menopausal status, n (%) [a]				
Pre/perimenopausal	38 (18.0)	47 (22.2)	31 (29.2)	38 (23.8)
Postmenopausal	155 (73.5)	147 (69.3)	66 (62.3)	114 (71.3)
Unknown	18 (8.5)	18 (8.5)	9 (8.5)	8 (5.0)
ECOG PS, n (%)				
0	135 (63.7)	134 (62.6)	52 (48.6)	101 (63.1)
1	30 (14.2)	37 (17.3)	38 (35.5)	25 (15.6)

≥2	5 (2.4)	7 (3.3)	1 (0.9)	2 (1.3)
Unknown	42 (19.8)	36 (16.8)	16 (15.0)	32 (20.0)
Disease sites, n (%)				
Visceral metastasis	212 (100.0)	214 (100.0)	107 (100.0)	160 (100.0)
Liver metastasis	0	72 (33.6)	0	73 (45.6)
Bone-only metastasis	105 (49.5)	0	50 (46.7)	0
DFI, n (%) [b]				
<24 months	21 (9.9)	18 (8.4)	9 (8.4)	14 (8.8)
≥24 months	140 (66.0)	138 (64.5)	68 (63.6)	97 (60.6)
TFI, n (%) [c]				
De novo metastasis/others	55 (25.9)	56 (26.2)	27 (25.2)	48 (30.0)
≥ 12 months	102 (48.1)	93 (43.5)	46 (43.0)	59 (36.9)
< 12 months	41 (19.3)	46 (21.5)	20 (18.7)	34 (21.3)
Symptoms at the start of palbociclib, n (%) [d]				
Yes	94 (44.3)	97 (45.3)	68 (63.6)	95 (59.4)
No	110 (51.9)	107 (50.0)	36 (33.6)	56 (35.0)
Unknown	8 (3.8)	10 (4.7)	3 (2.8)	9 (5.6)
Prior ET for (neo)adjuvant, n (%)	142 (67.0)	149 (69.6)	71 (66.4)	105 (65.6)
Prior CT for (neo)adjuvant, n (%)	117 (55.2)	100 (46.7)	57 (53.3)	68 (42.5)

CT : chemotherapy, DFI : disease-free interval (the time from the date of breast cancer surgery to the diagnosis date of recurrence), ECOG PS : Eastern Cooperative Oncology Group performance status, ET : endocrine therapy, TFI : treatment-free interval (the time from the end of adjuvant therapy to the diagnosis date of recurrence)

a The denominator is the number of female patients

b Percentage was calculated based on patients with disease stage other than “stage IV”. The patients without the date of breast cancer surgery were excluded from this calculation
c “Others” included patients who had surgery but did not undergo adjuvant therapy. The patients without the date of breast cancer surgery were excluded from this calculation
d Symptoms included bone pain, shortness of breath, coughing, headaches, dizziness, nausea, swelling around the neck and armpits, numbness in the limbs, abdominal bloating, and jaundice

Supplementary Table 4. Treatment pattern and dose modification of palbociclib in patients' presence/absence of visceral metastasis

	1L		2L	
	absence of visceral metastasis (n=212)	presence of visceral metastasis (n=214)	absence of visceral metastasis (n=107)	presence of visceral metastasis (n=160)
Initial palbociclib dose (mg/day)				
125	193 (91.0)	192 (89.7)	93 (86.9)	140 (87.5)
100	18 (8.5)	15 (7.0)	12 (11.2)	16 (10.0)
75	1 (0.5)	7 (3.3)	1 (0.9)	4 (2.5)
Other	0	0	1 (0.9)	0
Status of palbociclib administration [a]				
Ongoing	47 (22.2)	46 (21.5)	11 (10.3)	21 (13.1)
Discontinued	165 (77.8)	168 (78.5)	96 (89.7)	139 (86.9)
Reason for discontinuation of palbociclib [b, c]				
Disease progression	110 (66.7)	121 (72.0)	72 (75.0)	110 (79.1)
Adverse events	34 (20.6)	36 (21.4)	20 (20.8)	21 (15.1)
Other	24 (14.5)	15 (8.9)	6 (6.3)	11 (7.9)
Patients requiring dose reduction				
No	48 (22.6)	61 (28.5)	33 (30.8)	45 (28.1)
Yes	164 (77.4)	153 (71.5)	74 (69.2)	115 (71.9)
100 (mg/day)	57 (26.9)	49 (22.9)	26 (24.3)	45 (28.1)

75 (mg/day)	100 (47.2)	95 (44.4)	45 (42.1)	66 (41.3)
Other (mg/day)	7 (3.3)	9 (4.2)	3 (2.8)	4 (2.5)
Endocrine therapy in combination with palbociclib [d]				
Fulvestrant	124 (58.5)	116 (54.2)	84 (78.5)	122 (76.3)
Letrozole	75 (35.4)	87 (40.7)	19 (17.8)	24 (15.0)
Anastrozole	10 (4.7)	7 (3.3)	3 (2.8)	10 (6.3)
Exemestane	0	2 (0.9)	2 (1.9)	1 (0.6)
Tamoxifen	4 (1.9)	3 (1.4)	0	3 (1.9)

[a]Data cutoff date was February 16, 2024

[b]Percentage was calculated with patients who discontinued palbociclib.

[c]The different reasons for palbociclib discontinuation in the same patient were counted in the respective group.

[d]The different endocrine therapies used in the same patient within the same treatment line were counted in the respective group.

Supplementary Table 5. Demographics and clinical characteristics of patients' presence/absence of liver metastasis

	1L		2L	
	absence of liver metastasis (n=354)	presence of liver metastasis (n=72)	absence of liver metastasis (n=194)	presence of liver metastasis (n=73)
Age category (years), n (%)				
≤49	63 (17.8)	25 (34.7)	46 (23.7)	14 (19.2)
50–64	152 (42.9)	26 (36.1)	71 (36.6)	30 (41.1)
65–74	101 (28.5)	17 (23.6)	48 (24.7)	18 (24.7)
≥75	38 (10.7)	4 (5.6)	29 (14.9)	11 (15.1)
Sex, n (%)				
Male	3 (0.8)	0	1 (0.5)	0
Female	351 (99.2)	72 (100.0)	193 (99.5)	73 (100.0)
Menopausal status, n (%) [a]				
Pre/perimenopausal	65 (18.5)	20 (27.8)	51 (26.4)	18 (24.7)
Postmenopausal	253 (72.1)	49 (68.1)	129 (66.8)	51 (69.9)
Unknown	33 (9.4)	3 (4.2)	13 (6.7)	4 (5.5)
ECOG PS, n (%)				
0	222 (62.7)	47 (65.3)	108 (55.7)	45 (61.6)
1	52 (14.7)	15 (20.8)	54 (27.8)	9 (12.3)
≥2	10 (2.8)	2 (2.8)	3 (1.5)	0

Unknown	70 (19.8)	8 (11.1)	29 (14.9)	19 (26.0)
Disease sites, n (%)				
Visceral metastasis	142 (40.1)	72 (100.0)	87 (44.8)	73 (100.0)
Liver metastasis	0	72 (100.0)	0	73 (100.0)
Bone-only metastasis	105 (29.7)	0	50 (25.8)	0
DFI, n (%) [b]				
<24 months	28 (7.9)	11 (15.3)	13 (6.7)	10 (13.7)
≥24 months	233 (65.8)	45 (62.5)	122 (62.9)	43 (58.9)
TFI, n (%) [c]				
De novo metastasis/others	95 (26.8)	16 (22.2)	56 (28.9)	19 (26.0)
≥ 12 months	154 (43.5)	41 (56.9)	69 (35.6)	36 (49.3)
< 12 months	74 (20.9)	13 (18.1)	44 (22.7)	10 (13.7)
Symptoms at the start of palbociclib, n (%) [d]				
Yes	152 (42.9)	39 (54.2)	117 (60.3)	46 (63.0)
No	188 (53.1)	29 (40.3)	69 (35.6)	23 (31.5)
Unknown	14 (4.0)	4 (5.6)	8 (4.1)	4 (5.5)
Prior ET for (neo)adjuvant, n (%)	236 (66.7)	55 (76.4)	124 (63.9)	52 (71.2)
Prior CT for (neo)adjuvant, n (%)	176 (49.7)	41 (56.9)	90 (46.4)	35 (47.9)

CT : chemotherapy, DFI : disease-free interval (the time from the date of breast cancer surgery to the diagnosis date of recurrence), ECOG PS : Eastern Cooperative Oncology Group performance status, ET : endocrine therapy, TFI : treatment-free interval (the time from the end of adjuvant therapy to the diagnosis date of recurrence)

a The denominator is the number of female patients

b Percentage was calculated based on patients with disease stage other than “stage IV”. The patients without the date of breast cancer surgery were excluded from this calculation

c “Others” included patients who had surgery but did not undergo adjuvant therapy. The patients without the date of breast cancer surgery were excluded from this calculation

d Symptoms included bone pain, shortness of breath, coughing, headaches, dizziness, nausea, swelling around the neck and armpits, numbness in the limbs, abdominal bloating, and jaundice

Supplementary Table 6. Demographics and clinical characteristics of patients presence/absence of liver metastasis

	1L		2L	
	absence of liver metastasis (n=354)	presence of liver metastasis (n=72)	absence of liver metastasis (n=194)	presence of liver metastasis (n=73)
Initial palbociclib dose (mg/day)				
125	321 (90.7)	64 (88.9)	168 (86.6)	65 (89.0)
100	28 (7.9)	5 (6.9)	22 (11.3)	6 (8.2)
75	5 (1.4)	3 (4.2)	3 (1.5)	2 (2.7)
Other	0	0	1 (0.5)	0
Status of palbociclib administration [a]				
Ongoing	81 (22.9)	12 (16.7)	28 (14.4)	4 (5.5)
Discontinued	273 (77.1)	60 (83.3)	166 (85.6)	69 (94.5)
Reason for completion/discontinuation of palbociclib [b, c]				
Disease progression	58 (21.2)	12 (20.0)	32 (19.3)	9 (13.0)
Adverse events	184 (67.4)	47 (78.3)	125 (75.3)	57 (82.6)
Other	36 (13.2)	3 (5.0)	13 (7.8)	4 (5.8)
Patients requiring dose reduction				
No	91 (25.7)	18 (25.0)	53 (27.3)	25 (34.2)
Yes	263 (74.3)	54 (75.0)	141 (72.7)	48 (65.8)
100 (mg/day)	86 (24.3)	20 (27.8)	48 (24.7)	23 (31.5)
75 (mg/day)	162 (45.8)	33 (45.8)	86 (44.3)	25 (34.2)

Other (mg/day)	15 (4.2)	1 (1.4)	7 (3.6)	0
Endocrine therapy in combination with palbociclib [d]				
Fulvestrant	198 (55.9)	42 (58.3)	153 (78.9)	53 (72.6)
Letrozole	139 (39.3)	23 (31.9)	30 (15.5)	13 (17.8)
Anastrozole	11 (3.1)	6 (8.3)	8 (4.1)	5 (6.8)
Exemestane	1 (0.3)	1 (1.4)	3 (1.5)	0
Tamoxifen	6 (1.7)	1 (1.4)	1 (0.5)	2 (2.7)

[a]Data cutoff date was February 16, 2024.

[b]Percentage was calculated with patients who discontinued palbociclib.

[c]The different reasons for palbociclib discontinuation in the same patient were counted in the respective group.

[d]The different endocrine therapies used in the same patient within the same treatment line were counted in the respective group.

Supplementary Figure 1. Real-world A) PFS B) OS and C) CFS of palbociclib plus ET as 1L and 2L treatment in patients with ABC who started palbociclib 125 mg/day.

1L, first-line; 2L, second-line; ABC, advanced breast cancer; CFS, chemotherapy-free survival; CI, confidence interval; ET, endocrine therapy; NE, not evaluated; OS, overall survival; PFS, progression-free survival

A)

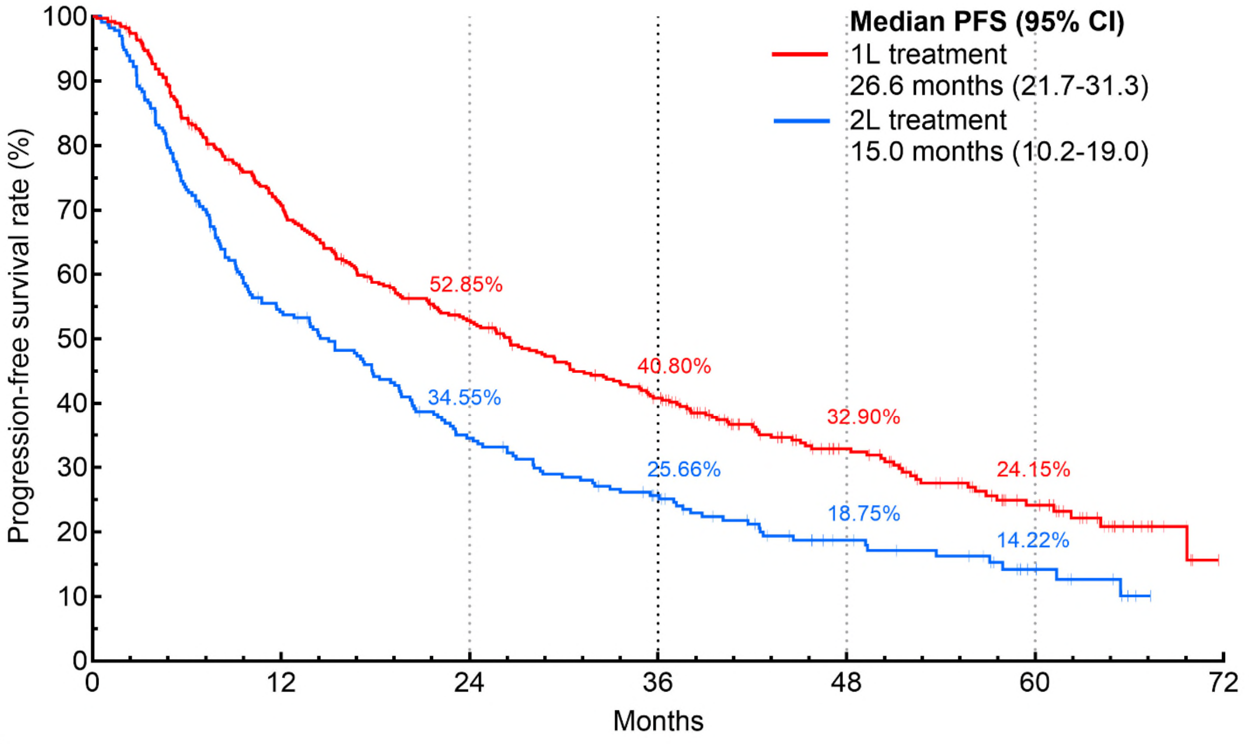

Population at risk							
1L treatment	385	258	185	134	66	31	0
2L treatment	233	171	75	49	24	10	-

B)

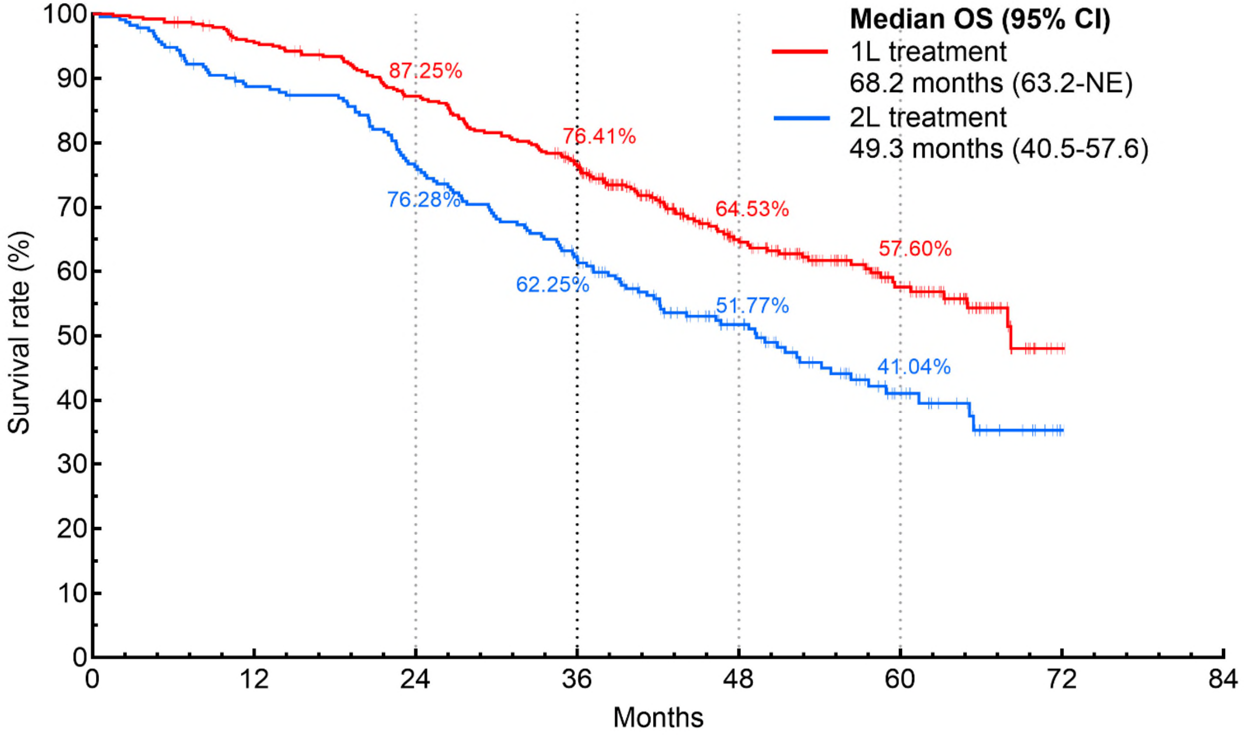

Population at risk							
1L treatment	385	361	325	274	150	78	2	0
2L treatment	233	201	171	132	77	31	1	0

C)

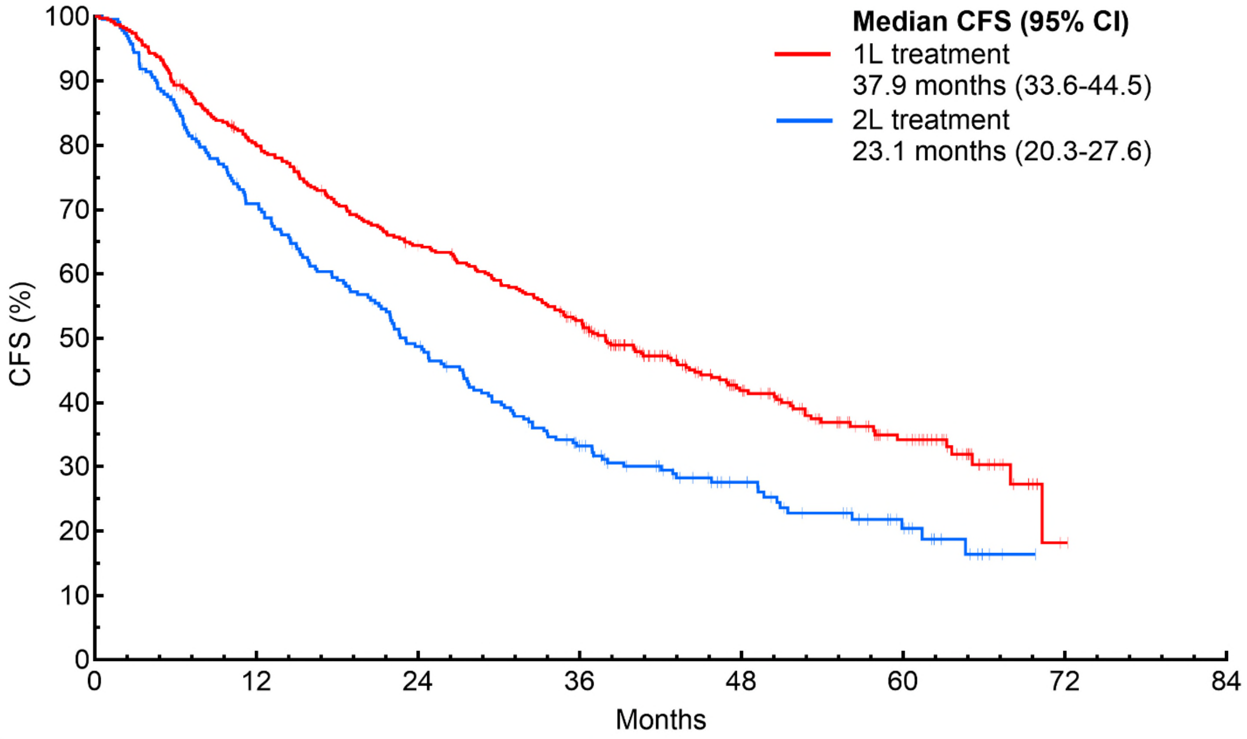

Population at risk

1L treatment	385	302	240	190	96	46	1	0
2L treatment	233	161	109	67	37	15	0	-

Supplementary Figure 2. Subgroup analysis of OS with palbociclib plus ET as 1L and 2L treatment in patients with ABC stratified by A) presence/absence of visceral metastasis B) presence/absence of liver metastasis C) presence/absence of bone only metastasis and D) treatment-free interval.1L, first-line; 2L, second-line; ABC, advanced breast cancer; CI, confidence interval; ET, endocrine therapy; NR, not reached; NE, not evaluated; OS, overall survival

A)

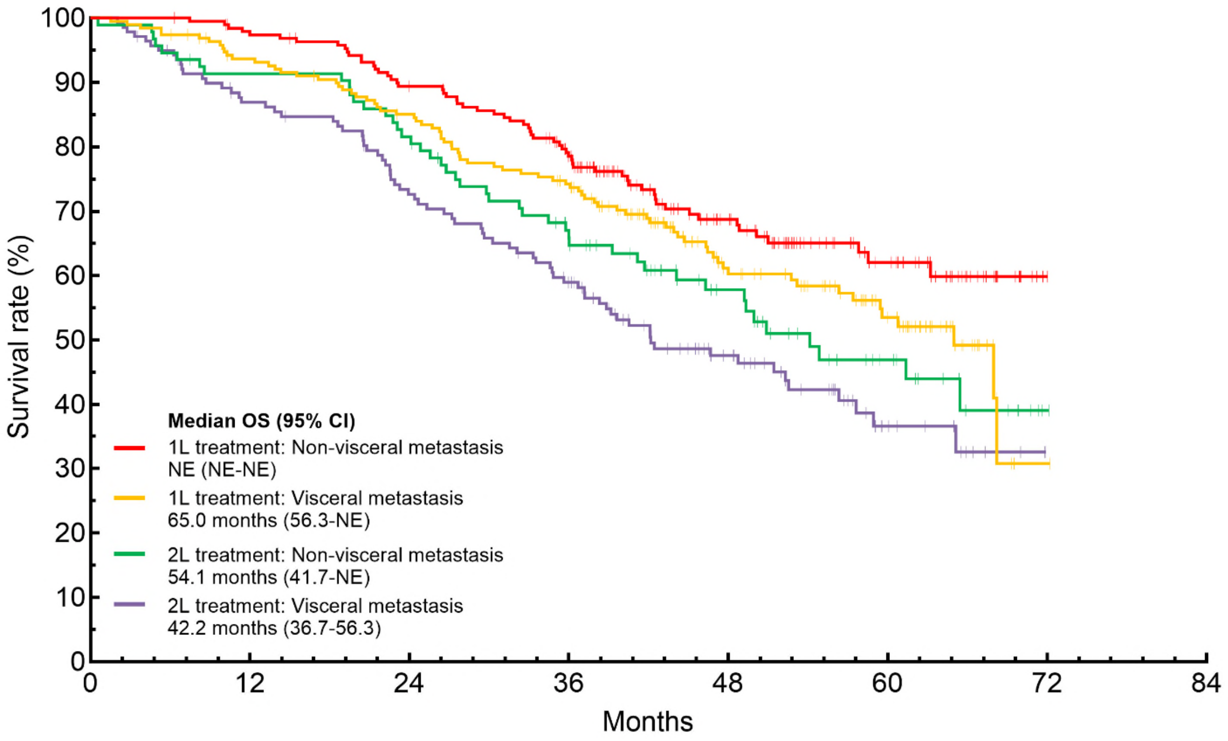

Population at risk

1L non-visceral metastasis	193	186	168	139	80	38	1	0
1L visceral metastasis	192	175	157	135	70	40	1	0
2L non-visceral metastasis	93	84	75	57	35	18	1	0
2L visceral metastasis	140	117	96	75	42	13	0	-

B)

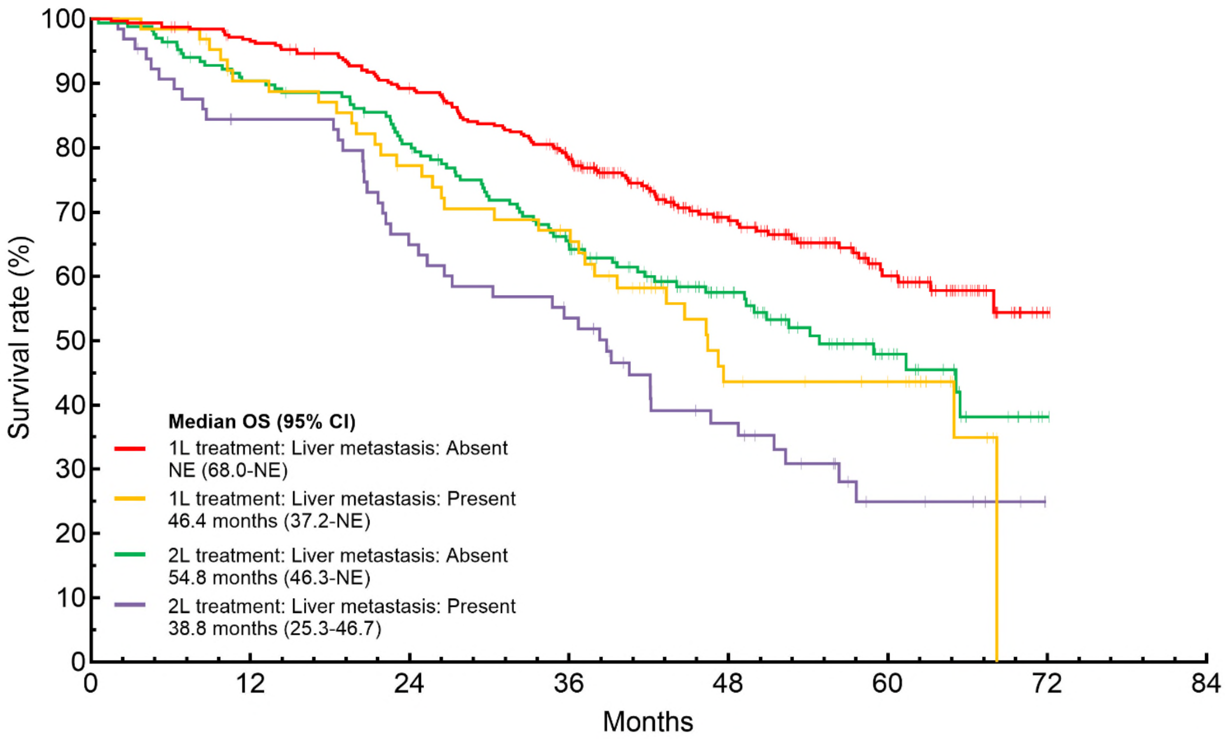

Population at risk

1L Liver metastasis: Absent	321	306	279	235	133	64	2	0
1L Liver metastasis: Present	64	55	46	39	17	14	0	-
2L Liver metastasis: Absent	168	149	131	100	58	24	1	0
2L Liver metastasis: Present	65	52	40	32	19	7	0	-

C)

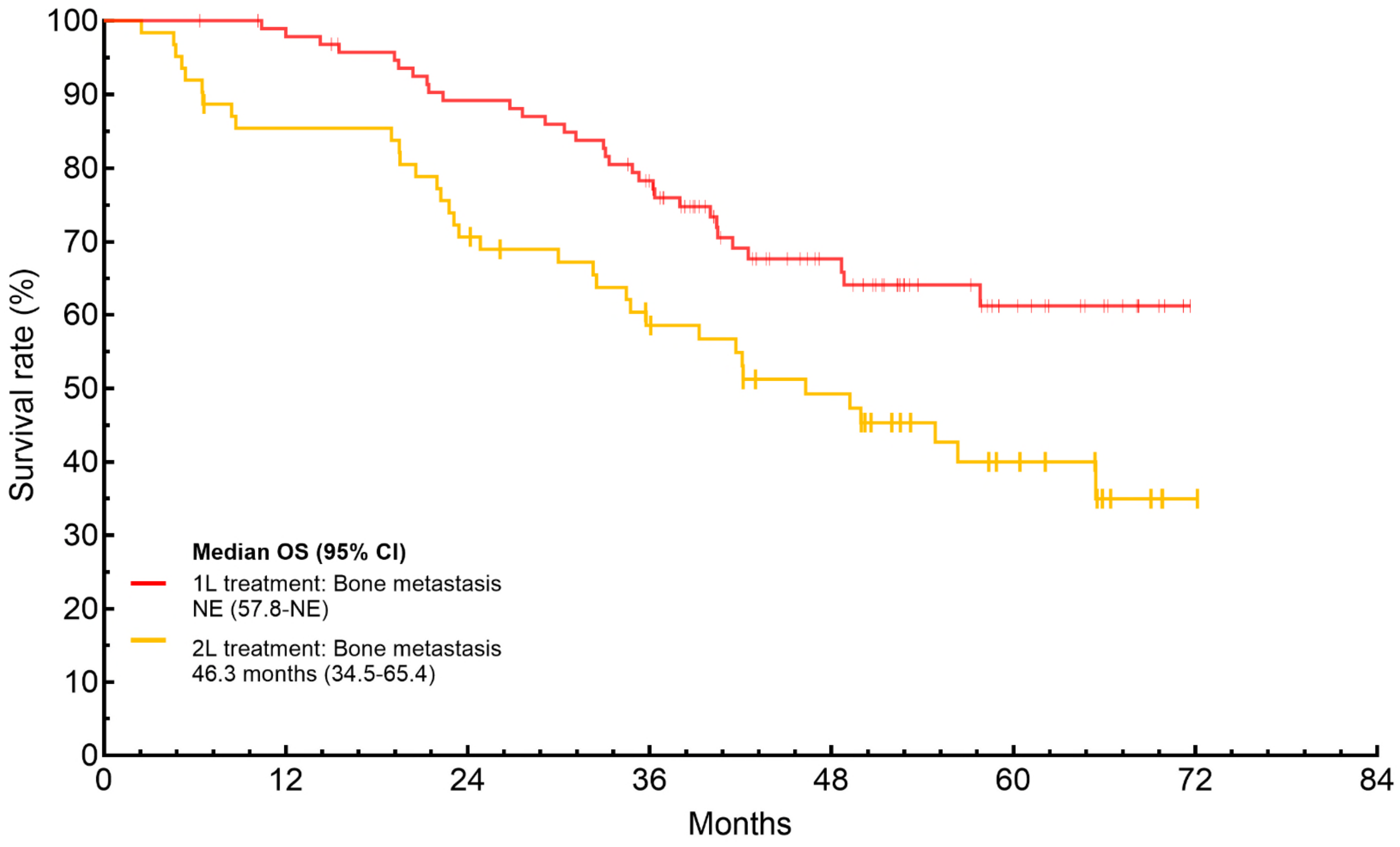

**Population at risk**								
1L Bone metastasis	96	93	82	69	38	17	0	-
2L Bone metastasis	62	52	43	33	25	12	1	-

D)

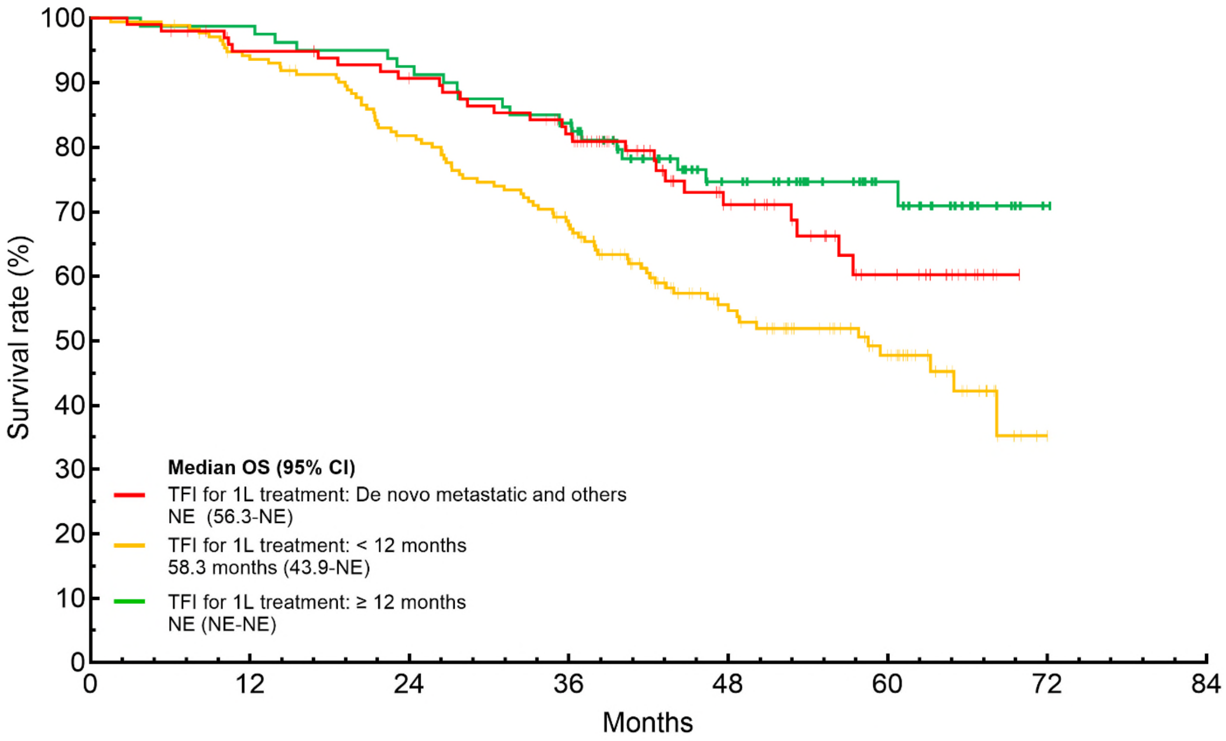

Population at risk

	0	12	24	36	48	60	72	84
TFI for 1L treatment: De novo metastatic and others	100	91	86	74	36	17	0	-
TFI for 1L treatment: < 12 months	174	161	137	109	61	33	1	0
TFI for 1L treatment: ≥ 12 months	80	79	74	66	38	20	1	0
